# A witches' broom phytoplasma effector induces stunting by stabilizing a bHLH transcription factor in *Ziziphus jujuba* plants

**DOI:** 10.1111/nph.70172

**Published:** 2025-05-09

**Authors:** Shuang Yang, Amelia H. Lovelace, Yi Yuan, Haizhen Nie, Weikai Chen, Yi Gao, Wenhao Bo, Dawn H. Nagel, Xiaoming Pang, Wenbo Ma

**Affiliations:** ^1^ State Key Laboratory of Tree Genetics and Breeding, National Engineering Research Center of Tree Breeding and Ecological Restoration, Key Laboratory of Genetics and Breeding in Forest Trees and Ornamental Plants, Ministry of Education, College of Biological Sciences and Biotechnology Beijing Forestry University Beijing 100083 China; ^2^ The Sainsbury Laboratory, Norwich Research Park Norwich NR4 7UH UK; ^3^ Department of Botany and Plant Sciences University of California Riverside Riverside CA 92521 USA

**Keywords:** bHLH transcription factor, effector, jujube witches' broom disease, phytoplasma, plant–pathogen interaction

## Abstract

- Phytoplasmas are specialized phloem‐limited bacteria that cause diseases on various crops, resulting in significant agricultural losses. This research focuses on the jujube witches' broom (JWB) phytoplasma and investigates the host‐manipulating activity of the effector SJP39.
- We found that SJP39 directly interacts with the plant transcription factor bHLH87 in the nuclei. SJP39 stabilizes the bHLH87 homologs in *Arabidopsis thaliana* and jujube, leading to growth defects in the plants.
- Transcriptomic analysis indicates that SJP39 affects the gibberellin (GA) pathway in jujube. We further demonstrate that ZjbHLH87 regulates GA signaling as a negative regulator, and SJP39 enhances this regulation.
- The research offers important insights into the pathogenesis of JWB disease and identified SJP39 as a virulence factor that can contribute to the growth defects caused by JWB phytoplasma infection. These findings open new opportunities to manage JWB and other phytoplasma diseases.

## Introduction

Jujube (*Ziziphus jujuba* Mill.) is one of the longest cultivated perennial fruit crops that originated in China (Li *et al*., 2024) with significant impacts on culture and economy. The jujube industry is currently facing an unprecedented threat due to the jujube witches' broom (JWB) disease, which affects main jujube cultivation areas in China (Guo *et al*., 2023). Diseased jujube trees exhibit a spectrum of symptoms including witches' broom, phyllody, leaf yellowing, and stunting trees (Bertaccini, 2022; Guo *et al*., 2023). Infected trees typically succumb to the disease within 2–3 yr (Zhou *et al*., 2021). Economic losses caused by JWB are not only due to reduced yield, fruit quality, and tree longevity but also due to substantial costs for disease control and management measures (J. Wang *et al*., 2018; Yang *et al*., 2023). Thus, a deep understanding of the virulence mechanisms underlying JWB development is crucial for developing effective disease control strategies and to safeguard the global jujube industry.

Jujube witches' broom is caused by phytoplasma infection. Phytoplasmas are obligate bacterial pathogens that colonize phloem sieve elements of infected plants through insect transmission (Ye *et al*., 2017). Phytoplasmas undergo a life cycle that alternates between plant hosts and specific insects (Wei *et al*., 2004). Insects become infected by acquiring phytoplasmas from the phloem of diseased plants. Once the bacteria invade the insect's salivary glands, they can be transferred to the phloem of healthy plants during feeding (Namba, 2019). These bacteria lack a cell wall and possess a highly reduced genome, reflecting their lifestyle as obligate pathogens (Oshima *et al*., 2013; Guo *et al*., 2023). The phytoplasma responsible for JWB belongs to the 16SrV‐B subgroup (Xue *et al*., 2023). The primary insect vector for JWB phytoplasma transmission is the leafhopper *Hishimonus sellatus* (Jung *et al*., 2003). In addition, JWB can also spread through grafting practices (Lee *et al*., 2012; Ye *et al*., 2017). Currently, few chemical or biological control methods exist for JWB disease management. Previous studies have demonstrated that techniques such as shoot tip culture to produce pathogen‐free propagations (Wang *et al*., 2009; Namba, 2019) and injecting tetracycline derivatives into infected stems or seeds can reduce phytoplasma infection in plant explants (Askari *et al*., 2011; Bertaccini, 2022). However, these management measures are costly, laborious, and time‐consuming. While there is an urgent need to develop novel approaches to accomplish sustainable resistance to JWB in jujube, achieving this goal requires a comprehensive understanding of the disease and the phytoplasma pathogen.

During the infection process, phytoplasma secretes a variety of effector proteins via the sec‐dependent pathway (Sugio *et al*., 2011b). These effectors are crucial for phytoplasma colonization, insect transmission, and disease symptom development. To date, *c*. 20 phytoplasma effectors have been experimentally studied, although the number of predicted effector proteins is much larger (Carreón‐Anguiano *et al*., 2023). The best‐studied effectors are encoded in the strain Aster yellows witches' broom (AY‐WB; or *Ca* Phytoplasma asteris), which infects the model plant *Arabidopsis thaliana* (Sugio *et al*., 2011a). Aster yellows witches' broom was predicted to secrete 56 *s*ecreted *A*Y‐WB *p*roteins (SAPs) as putative effectors (Bai *et al*., 2009). Some of these SAPs are mobile and affect host cellular processes in adjacent companion cells or distal meristem tissue (MacLean *et al*., 2011). So far, all characterized SAPs (SAP11, SAP54, and SAP05) have been shown to target plant transcription factors (TFs), manipulating host cells by reprograming their transcriptome (Lakhanpaul *et al*., 2023). This transcription reprograming facilitates not only colonization in the plant tissue but also insect transmission (Orlovskis *et al*., 2023). For example, SAP11 interacts with and destabilizes class II TCP (TEOSINTE BRANCHED1/CYCLOIDEA/PROLIFERATING CELL FACTOR) host TFs, resulting in leaf crinkling and stem proliferation. Homologs of SAP11 from maize bushy stunt phytoplasma (MBSP), wheat blue dwarf phytoplasma (WBD), and apple proliferation (AP) phytoplasma – referred to as SAP11_MBSP_, SAP11_WBD_, and SAP11_AP_, respectively – also interact with TCP TFs. These interactions lead to significant alterations in leaf and shoot architecture (N. Wang *et al*., 2018; Lakhanpaul *et al*., 2023). *S*ecreted *A*Y‐WB *p*roteins can induce additional developmental anomalies such as phyllody and bolting (Orlovskis & Hogenhout, 2016; Iwabuchi *et al*., 2020). For example, SAP54_AY‐WB_ degrades MADS‐box TFs in transgenic *A. thaliana*, resulting in the formation of leafy flowers (MacLean *et al*., 2011).

Homologs of SAPs in JWB phytoplasma have also been studied. These effectors are named *s*ecreted *J*WB *p*roteins (SJPs). The SAP11_AY‐WB_ homologs SJP1 and SJP2 promote lateral bud outgrowth by destabilizing the jujube protein ZjBRC1 and regulating auxin efflux (Zhou *et al*., 2021). The SAP54_AY‐WB_ homolog SJP3 induces pistil reversion partly through interaction with MADS‐box TF SHORT VEGETATIVE PHASE 3 (Deng *et al*., 2021, 2024). Despite these insights, many SJPs remain uncharacterized.

Here, we identified and characterized the JWB phytoplasma effector, SJP39 (AYJ01459.1), which is the most highly expressed SJP during jujube infection. SJP39 interacts with the TF ZjbHLH87 (XP_015866259.1) and stabilizes its accumulation. Overexpression of *SJP39* and *ZjbHLH87* in transgenic jujube led to stunted growth, mirroring JWB symptoms, potentially through inhibiting gibberellin (GA) signaling. This finding uncovers a molecular mechanism underlying JWB pathogenesis and opens new avenues for enhancing jujube resistance to JWB.

## Materials and Methods

### Plant materials and bacterial strains

*Arabidopsis thaliana* plants were grown in a glasshouse under either long‐day (16 h light : 8 h dark) or short‐day (10 h light : 14 h dark) conditions at 22°C, with plant age calculated from the date seeds were transferred to growth chambers following stratification. *Atbhlh87* mutant (SALK_066339) line was obtained from the Nottingham Arabidopsis Stock Centre resource center. *Nicotiana benthamiana* plants were grown in soil under a 16 h light : 8 h dark cycle at 22°C in a growth chamber. Jujube ‘Jingzao 39’ seedlings were grown in subculture media (Murashige and Skoog (MS) medium) supplemented with 0.3 mg l^−1^ 6‐butyric acid (6‐BA), 0.2 mg l^−1^ indolebutyric acid (IBA), and 0.1 mg l^−1^ gibberellic acid (GA3)) and managed under long‐day (14 h : 10 h (light : dark)) conditions at 25 ± 1°C.

Diseased jujube trees (*Ziziphus jujuba* Mill.) were sampled from orchards in Changping, Beijing, and Cangzhou, Hebei. Branches and roots were collected from ‘Suanzao’ (*Z. jujuba* Mill. var. *spinosa*) and ‘Jinsixiaozao’ (*Z. jujuba* Mill. ‘Jinsixiaozao’) trees exhibiting symptoms of JWB. Three independent samples were taken from each cultivar and tissue type.

### *Agrobacterium*‐mediated transformation in *N. benthamiana*

Agroinfiltration‐based transient gene expression in *N. benthamiana* leaves was performed as described with minor modifications (MacLean *et al*., 2014). Briefly, TFs‐Myc and GFP‐SJP39^32–114^ (the mature SJP39 protein excluding the N‐terminal secretion signal peptide (SP), 1–31 aa), GFP‐SJP39^32–77^ (containing a predicted coiled‐coil domain, 48–77 aa), or GFP were expressed in *N. benthamiana* leaves for checking protein abundance. Proteins were separated by 12% SDS‐PAGE and detected using anti‐GFP (1 : 2000; Merck, Rahway, New Jersey, USA) and anti‐Myc (1 : 2000, Merck) antibodies.

### Genome analysis of JWB phytoplasma for effector prediction

The JWB phytoplasma genome (GenBank assembly accession GCA_003640545.1) was analyzed following the method described by Bai *et al*. (2009) to identify candidate effectors. The presence of N‐terminal SP within predicted open reading frames (ORFs) was assessed using the signalp program across three versions: signalp v.3.0 (https://services.healthtech.dtu.dk/services/SignalP‐3.0/), signalp v.4.0 (http://www.cbs.dtu.dk/services/SignalP‐4.0/), and signalp v.5.0 (https://services.healthtech.dtu.dk/services/SignalP‐5.0/) as described by Petersen *et al*. (2011). Open reading frames predicted to encode transmembrane domains were identified using tmhmm v.2.0 (http://www.cbs.dtu.dk/services/TMHMM/). Open reading frames containing transmembrane domains were excluded from further analysis. The presence of a nuclear localization signal (NLS) was determined using PSORT and PredictNLS (Cokol *et al*., 2000) programs. SJP15 and SJP43, identified as effectors by Deng *et al*. (2021), were included in this study. The list of candidates SJP effectors, signalp, and results are provided in Supporting Information Table S1.

### Virus‐induced virulence effector assay

The virus‐induced virulence effector assay was conducted as previously described (Shi *et al*., 2020). Briefly, PCR products of SJP39 and SJP37 (lacking the SPs) were ligated into the pGR107 vector, which is driven by the strong constitutive Cauliflower Mosaic Virus 35S (CaMV 35S) promoter and contains the complete potato virus X (PVX) genome. The resulting construct was introduced into GV3101 and subsequently infiltrated into the leaves of 10‐d‐old *N. benthamiana* plants. Viral symptoms were monitored and photographed at 14 d postinoculation (dpi). Leaves inoculated for 2–6 dpi were harvested, and total protein was extracted as previously described (Li *et al*., 2023). Viral titers were examined by western blotting using an antibody specific to the PVX coat protein (CP).

### Confocal microscopy

*SJP39* and *SJP39* ^*32–77*^ were cloned into the pYBA1152 vector, in which *SJP39* and *GFP‐SJP39* ^*32–77*^ are fused with N‐terminal GFP. For evaluating the impact of SJP39 on the nuclear accumulation of ZjbHLH87, the coding sequence of *ZjbHLH87* was cloned into pYBA1152 vector, whereas mature *SJP39* and *SJP39* ^*32–77*^ were cloned into the vector pGDR‐DsRed. GFP‐ZjbHLH87 and H2B‐CFP were co‐expressed with pGDR‐DsRed, DsRed‐SJP39, or DsRed‐SJP39^32–77^ in four replicate *N. benthamiana* plants. After 3  dpi, fluorescence was visualized using a confocal laser scanning microscope as described above. The percent gain was maintained for GFP channels at 25%. Four images were taken from each sample, and nuclear GFP intensity was quantified using imagej (Brazill *et al*., 2018). For each image, the nuclear CFP channel was converted to binary, and nuclei were defined using the Analyse Particle feature (> 10‐micron, 0–1 circularity). Nuclei were added to the Region of interest (ROI) manager, and GFP intensities were measured for each nucleus in the images. Each experiment was repeated three times independently. Plasmids used in this study are listed in Table S2.

### Yeast two‐hybrid screening

In the yeast two‐hybrid (Y2H) screen, the *SJP39* gene, excluding its secretory SP, was cloned into the pDEST32 plasmid using the Gateway system (Thermo Fisher Scientific, Waltham, MA, USA) and screened against a 1956 *A. thaliana* TF library (pDEST22‐AtTF), as previously described (de Folter & Immink, 2011; Pruneda‐Paz *et al*., 2014). For Y2H assays, *SJP39*, along with its truncated forms *SJP39* ^*32–77*^ and *SJP39* ^*78–114*^ (containing the C‐terminal region of SJP39 without the predicted CC domain), was cloned into pDEST32, whereas the coding sequences of *AtbHLH87* and *ZjbHLH87* were cloned into pDEST22. The recombinant vectors were co‐transformed into the AH109 strain, and yeast cells containing both bait and prey vectors were grown on an leucine‐tryptophan‐histidine triple‐selective medium (SD‐LTH) medium supplemented with 10 mM 3‐AT at 28°C for 3–5 d. Empty pDEST22/32 vectors and the pDEST22‐AtTCP13 (AT3G02150)/pDEST32‐SAP11 combination were used as negative and positive controls, respectively (Sugio *et al*., 2011a).

### Phylogenetic analyses

For the phylogenetic analysis of bHLH87, the sequences from the VIIIb subfamily of the bHLH TF family (Gao & Dubos, 2023) in *A. thaliana* and jujube were obtained from the Plant Transcription Factor Database (PlantTFDB, https://planttfdb.gao‐lab.org/). Multiple sequence alignment was performed, and a phylogenetic tree was constructed using mega X (Tamura *et al*., 2021). For the phylogenetic analysis of *Candidatus* phytoplasma, the TimeTree of Life website (https://timetree.org/) was used (Kumar *et al*., 2017).

### *In vitro* pull‐down assay

Mature *SJP39* and truncates *SJP39* ^*32–77*^ and *SJP39* ^*78–114*^ were cloned into the pGEX4T‐1 vector containing the glutathione S‐transferase (GST) tag, whereas *ZjbHLH87* and *AtbHLH87* were cloned into the pET‐30a vector containing histidine (His) tag. These constructs, along with empty vectors, were expressed in *Escherichia coli* BL21. Purified GST, GST‐SJP39, GST‐SJP39^32–77^, GST‐SJP39^78–114^, His‐ZjbHLH87, and His‐AtbHLH87 fusion proteins were incubated with glutathione agarose (Thermo Scientific) at 4°C for 3 h. After incubation, the beads were washed four times with wash buffer (20 mM Tris–HCl, pH 7.4, 1 mM EDTA, 200 mM NaCl, and 1 mM Dithiothreitol (DTT)), and proteins were eluted using elution buffer (20 mM Tris–HCl, pH 7.4, 200 mM NaCl, 1 mM DTT, and 10 mM glutathione). Both input and pull‐down samples were separated by 12% SDS‐PAGE and analyzed by immunoblotting with anti‐GST antibody (1 : 2000; TransGen Biotech, Beijing, China) or anti‐His antibody (1 : 2000; Thermo Scientific).

### Co‐immunoprecipitation assay

For co‐immunoprecipitation (Co‐IP) assays, the coding sequences of mature *SJP39*, *SJP39* ^*32–77*^, and *ZjbHLH87*, *AtbHLH87* were cloned into pYBA1152 and pCAMBIA‐1300 vectors, respectively. The resulting constructs were transformed into *Agrobacterium tumefaciens* GV3101 and co‐infiltrated into *N. benthamiana* leaves. After 2 dpi, *c*. 2 g of leaf material was ground into a fine powder in liquid nitrogen and homogenized in 4 ml of extraction buffer containing 2% Polyvinylpolypyrrolidone (PVPP), 10% glycerol, 50 mM Tris (pH 7.5), 1 mM EDTA (pH 8.0), 150 mM NaCl, 0.1% NP‐40, 1% (v/v) cOmplete‐EDTA‐free protease inhibitor cocktail (Sigma), 25 mM MG132 (Merck), 1 mM phenylmethylsulfonyl fluoride (PMSF), and 1 mM DTT. Total protein extracts were incubated with 10 μl of GFP‐Trap magnetic agarose beads (gtma‐400; Ychromotek, Bavaria, Germany) for 1.5 h at 4°C. The beads were washed three times with wash buffer (extraction buffer without PVPP) and resuspended with 30 μl wash buffer and boiled for 5 min in10 μl 4 × SDS loading buffer containing 10 mM DTT. Both input and immunoprecipitated samples were separated by 12% SDS‐PAGE and detected using anti‐GFP (1 : 2000; Merck) and anti‐Myc (1 : 2000; Merck) antibodies.

### MG132 treatment

For chemical treatments, MG132 and cycloheximide (CHX) (Sigma) were dissolved in dimethylsulfoxide (DMSO) and used at a final concentration of 100 μM. To analyze ZjbHLH87 degradation by the 26S proteasome, *N. benthamiana* plants were co‐infiltrated with the following combinations: ZjbHLH87‐Myc, ZjbHLH87‐Myc + GFP‐SJP39, and ZjbHLH87‐Myc + GFP‐SJP39^32–77^. Two dpi, leaves were treated with DMSO + CHX or MG132 + CHX at the indicated concentrations. Six hours after treatment, three leaf disks (1 cm^2^) were harvested. The samples were ground into a fine powder in liquid nitrogen and homogenized in 200 μl of Glucose‐Tris‐EDTA‐NaCl (GTEN) buffer containing 1 mM DTT and 1% (v/v) complete‐EDTA‐free protease inhibitor cocktail (Sigma). Proteins were separated by 12% SDS‐PAGE and analyzed by immunoblotting with anti‐GFP (1 : 2000; Merck) and anti‐Myc (1 : 2000; Merck) antibodies.

### Generation of transgenic *A. thaliana* and jujube lines

To generate transgenic plants overexpressing *SJP39* and *ZjbHLH87*, the *SJP39* gene was cloned into the pYBA1152 vector with an N‐terminal GFP tag, whereas *ZjbHLH87* was cloned into the pCAMBIA‐1300 vector with a C‐terminal Myc tag. Each gene construct, along with its corresponding empty vector control, was introduced into *A. tumefaciens* strain GV3101. All *A. thaliana* lines used were in the Columbia‐0 (Col‐0) background, and transgenic lines were produced using the *Agrobacterium*‐mediated floral dip method as described by Clough & Bent (1998). All transgenic *A. thaliana* lines utilized for experimentation were at the T3 generation. For overexpression of *SJP39* and *ZjbHLH87* in jujube, genes were cloned into the pYBA1152 vector with an N‐terminal GFP tag and subsequently transformed into *A. tumefaciens* strain GV3101, along with GFP as a control. All transgenic jujube lines were generated using the leaf disk method (Feng *et al*., 2010).

### RNA isolation and RT‐qPCR analysis

Total RNA was extracted from jujube transgenic lines using the FastPure Cell/Tissue Total RNA Isolation Kit V2 (Vazyme, Nanjing, China). For complementary DNA (cDNA) synthesis, 1 μg of the isolated RNA was utilized with the HiScript IV First‐Strand cDNA Synthesis Kit using the DNase I treatment to remove genomic DNA (Vazyme). Quantitative PCR was conducted with the HiScript II One Step qRT‐PCR SYBR Green Kit (Vazyme), following the reaction conditions and program outlined by Zhou *et al*. (2021). The relative expression levels were calculated using the 2^−ΔΔCt^ method (Livak & Schmittgen, 2001), with normalization against the *ZjACT1* gene (LOC107413530) for all jujube‐related samples and positive transgenic lines. A list of primers used in this study can be found in Table S3.

### RNA‐Seq analysis

Total RNA from infected jujube was extracted using the Omega Total RNA Extraction kit. Phytoplasma mRNA was enriched using the MICROBEnrich™ kit (Thermo Fisher Scientific) and Ribo‐Zero rRNA Removal kit (Epicentre, Madison, WI, USA), respectively. RNA quality and concentration were assessed with a Nanodrop 2000 spectrophotometer. Paired‐end 150‐bp sequencing was performed using the Illumina HiSeq™ 2500 platform.

Three 4‐wk‐old transgenic lines carrying either *GFP*, *SJP39*, or *ZjbHLH87*, were used for RNA‐Seq. RNA extraction was performed as above. RNA library construction and sequencing were conducted by NovaSeq (Beijing, China) using the DNBseq‐T7 platform, with 150‐bp paired‐end reads. The quality of the raw sequencing data was checked with fastqc v.0.11.9 (Andrews, 2010). Subsequent statistical analyses were performed using R. To ensure high‐quality sequences for mapping and downstream analyses, low‐quality reads and adapter had been trimmed by the sequencing facility before sequence delivery. RNA‐Seq reads were aligned with the indexed jujube cultivar Dongzao genome assembly (GCF_031755915.1) and analyzed using hisat2 v.2.2.1. The number of reads mapped to each gene was counted using the feature counts function of the rsubread package (Liao *et al*., 2019). The transgenic jujube data used for the analysis has been deposited into the ENA database (BioProject No. PRJEB81825). Differentially expressed genes (DEGs) were identified from gene counts for each sample using the deseq2 package v.1.30.1 from Bioconductor (Love *et al*., 2014). Differentially expressed genes were selected based on |log_2_‐fold change| > 2 that have an adjusted *P*‐value (*P*_adj_) below a false discovery rate cutoff of 0.05. Principal component analysis of rlog transformed read counts was performed for all samples using the plotPCA function in deseq2. The GO term analysis was conducted using the biocmanager package, viseago v.1.4.0. The custom GO terms for jujube were annotated using the Custom2GO and its annotation function. Enriched GO terms were identified from DEG sets using the entire genome as the background gene set using the ‘create_topGO data’ function. To test for significant enrichment, the classic algorithm and Fisher exact test were used with a cutoff *P*‐value of 0.05. Redundant‐enriched GO terms were identified using the REVIGO web server using the very small parameter (Supek *et al*., 2011).

Jujube genes identified belonging to the GA biosynthesis pathway based on the Kyoto Encyclopedia of Genes and Genomes pathway KEGG:zju00904 (Diterpenoid biosynthesis). Gibberellin‐responsive genes were identified based on their annotation to the GO term GO:0009739 (response to GA stimulus) and product description. Jujube genes identified belonging to the jasmonic acid (JA) biosynthesis pathway based on their annotation to the GO terms GO:0009694 (JA metabolic process) and GO:0009695 (JA biosynthetic process). JA signaling genes were identified based on their annotation to the GO terms GO:0009867 (JA‐mediated signaling pathway), GO:2000022 (regulation of JA‐mediated signaling pathway), and GO:0009753 (response to JA).

### Dual‐luciferase assay

For promoter cloning, genomic DNA from jujube was extracted using the DNeasy Plant Mini Kit (69 106; Qiangen). A 1021‐bp fragment containing the promoter of *ent‐kaurenoic acid oxidase* (*ZjKAO*, LOC107420337) and a 1106‐bp fragment containing the promoter of *GA‐regulated protein 11* (*ZjGRP11*, LOC107418476) were amplified by PCR and cloned into the pGreen II 0800‐LUC vector to drive expression of the firefly luciferase (LUC) gene as a reporter. Recombinant LUC vectors were individually transformed into GV3101 cells harboring the pSoup vector which constitutively expresses Renilla (Ren) LUC activity. Additional constructs, including AtbHLH87/ZjbHLH87‐Myc, pYBA1152, GFP‐SJP39, and GFP‐SJP39^32–77^, were also prepared. GV3101 cultures were combined and adjusted to a final OD_600_ of 0.5 in infiltration buffer and co‐infiltrated into *N. benthamiana* leaves. After 2 d, agroinfiltrated leaves were harvested, and LUC activity was measured using the Dual‐Glo Luciferase Assay System (E2920; Promega). Briefly, two leaf disks (4 mm in diameter) were collected in 2‐ml tubes, ground to a powder in liquid nitrogen, and 100 μl Passive Lysis System (PLS) buffer (E1941; Promega) was added. After centrifuging at 4°C for 10 min, followed by another 5‐min centrifugation, 75 μl of LUC assay reagent was mixed with an equal volume of supernatant in a 96‐well plate (CLS3922; Merk), and firefly luminescence was measured on a SpectraMax ID5 plate reader. Renilla luminescence was measured 10 min later using 75 μl of Stop & Glo reagent (Albert *et al*., 2021). Results were expressed as the ratio of firefly to Renilla luciferase (LUC/Ren) activity. This experiment was repeated three times.

### Gibberellin treatment

Stem segments (*c*. 1 cm in length) from jujube ‘Jingzao 39’ wild‐type (WT), *GFP* transgenic plants, *GFP‐SJP39* transgenic plants, and *GFP‐ZjbHLH87* transgenic plants were excised, with all leaves removed. These stem segments were placed on a MS medium supplemented with 0.3 mg l^−1^ 6‐BA and 0.2 mg l^−1^ IBA, with or without 2 mg l^−1^ GA3, and cultured for 4 wk under long‐day conditions (14 h light : 10 h dark) at 25 ± 1°C. After 4 wk, plant height was measured, and photographs were taken for documentation, and *c*. 4‐wk‐old *A. thaliana* plants expressing *GFP*, *GFP‐SJP39*, and *GFP‐SJP39* ^*32–77*^ were sprayed weekly with 100 μM GA3 for 3 wk (Debeaujon & Koornneef, 2000). Plant height was measured in the sixth week. This experiment was repeated three times.

### Statistical analysis

Statistical analysis was performed in prism 8.2. One‐way ANOVA was used to analyze experimental data with more than two experimental groups followed by Tukey's multiple comparisons test, and two‐tailed unpaired Student's *t*‐test was used for other data analysis.

## Results

### SJP39 is a highly expressed effector of JWB phytoplasma with plant‐manipulating activity

**Fig. 1:**
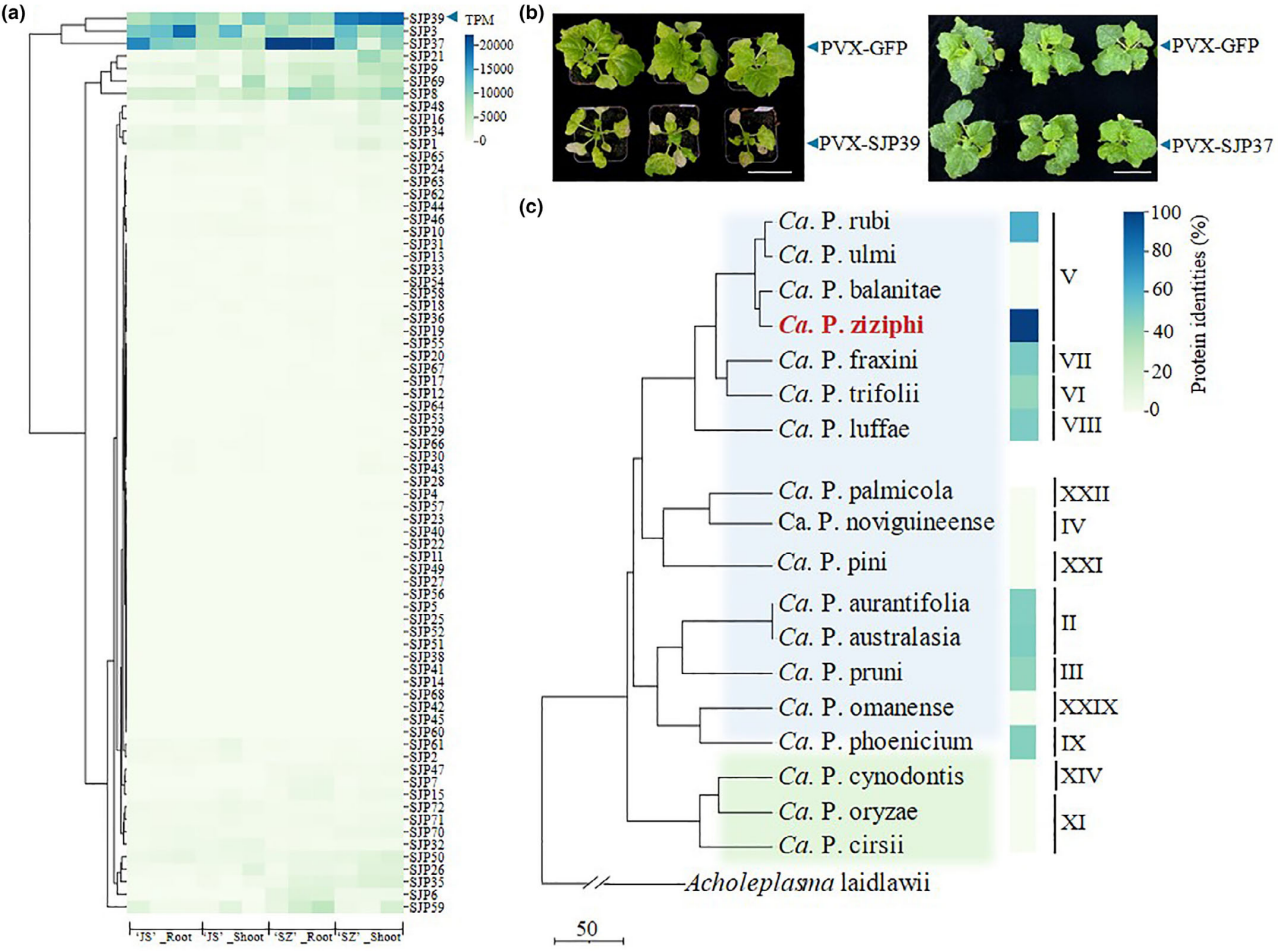
SJP39 is a putative effector produced by jujube witches’ broom (JWB) phytoplasma. (a) SJP39 is highly expressed in JWB‐infected jujube (*Ziziphus jujuba* Mill.). Expression profiles of predicted Sec‐dependent JWB proteins were determined by RNA‐seq in the root and shoot tissues of two jujube varieties ‘jin si xiao zao’ (‘JS’) and ‘suan zao’ (‘SZ’). (b) SJP39 enhanced necrosis after potato virus X (PVX) infection in *Nicotiana benthamiana*. Ten‐day‐old seedlings of *N. benthamiana* (*n* = 12) were inoculated with *Agrobacterium* carrying the vectors PVX‐GFP, PVX‐SJP37, or PVX‐SJP39 vectors. The images were taken at 14 d post agroinfiltration. This experiment was repeated three times with similar results. Bars, 5 cm. (c) SJP39 homologs are produced by multiple phytoplasmas. The figure displays a time‐calibrated phylogenetic tree (TimeTree) representing the evolutionary relationships among various *Candidatus* Phytoplasma (*Ca*. P.) species. Each branch corresponds to a species, and the tree scale (bottom left) indicates divergence time in millions of years (Myr), with 50 Myr as the reference. The 16S rRNA gene (16Sr) group assignments of representative phytoplasmas were determined by Davis *et al*. (2017). *Acholeplasma laidlawii* was used as the outgroup. The presence and absence of SJP39 homolog in each phytoplasma as well as their amino acid sequence similarity are indicated.

To identify potential virulence factors, we predicted secretion proteins, based on the presence of an N‐terminal SP and absence of transmembrane domains, from the genome of JWB phytoplasma (GenBank assembly accession GCA_003640545.1). This analysis revealed 72 SJP candidates (Table S1), including 43 that have been previously reported (Deng *et al*., 2021). The predicted SJPs were investigated for expression profiles in jujube by RNA‐Seq. Shoot and root tissues of infected ‘Suanzao’ (*Z. jujuba* Mill. var. spinosa) and ‘Jinsixiaozao’ (*Z. jujuba* Mill. ‘Jinsixiaozao’) were collected and analyzed. RNA‐Seq results show that SJP3, SJP37, and SJP39 exhibited the highest expression levels in jujube (Fig. 1a), suggesting that they may play a role in colonizing jujube plants. SJP3 has been previously characterized to disrupt pistil development in *A. thaliana* and *N. benthamiana* (Deng *et al*., 2021, 2024). SJP39 was highly expressed in shoot tissues in ‘Suanzao’, whereas SJP37 was highly expressed in the roots.

We further investigated SJP37 and SJP39 for potential plant‐manipulating activity in *N. benthamiana*. These two effectors were cloned into a binary PVX vector for *Agrobacterium*‐mediated transient expression. Seedlings infected with PVX‐SJP39 displayed chlorosis and necrosis on the inoculated leaves at 14 dpi and severe growth defects compared to plants infected with PVX‐GFP or PVX‐SJP37 (Figs 1b, S1a). The observed phenotype cannot be attributed to increased viral titers. In fact, the levels of PVX CP were lower in plants inoculated by PVX‐SJP39 compared to those inoculated with PVX‐GFP (Fig. S1b). Interestingly, expression of SJP39 using *Agrobacterium*‐mediated transient expression did not lead to a visible yellowing phenotype in *N. benthamiana* (Fig. S1c,d), indicating that SJP39 may enhance disease symptom development caused by PVX. Thus, it may function as a virulence factor and manipulate host plants.

According to the established classification scheme and a prior study on 16S rRNA gene phylogeny (Davis *et al*., 2017), ‘*Candidatus* Phytoplasma ziziphi (*Ca*. P. ziziphi)’ belongs to group 16SrV and is most closely related to ‘*Ca*. P. rubi’, ‘*Ca*. P. ulmi’, and ‘*Ca*. P. balanitae’ (Fig. 1c). To investigate the conservation of SJP39 in phytoplasmas, we searched for secreted proteins with sequence similarity across thirteen 16Sr groups known to infect diverse plant species. This analysis identified nine SJP39 homologs among 18 *Ca*. P. species, with protein identities ranging from 41.94% to 63.16%. These findings suggest that SJP39 may play a role in facilitating phytoplasma interaction with various plant hosts.

### SJP39 interacts with the bHLH87 transcription factor in plants

**Fig. 2:**
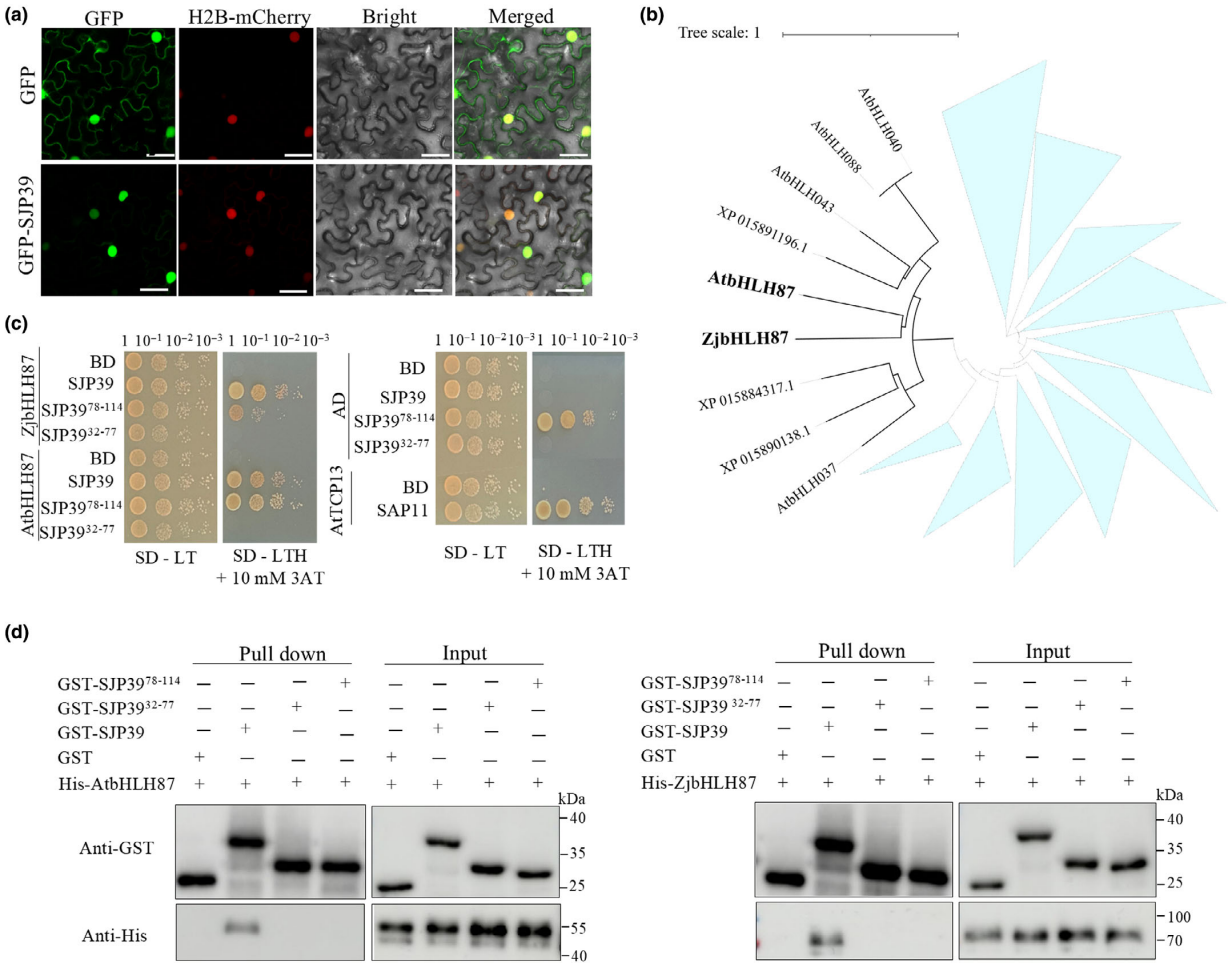
SJP39 interacts with the *Arabidopsis thaliana* transcription factor (TF) bHLH87 and its jujube (*Ziziphus jujuba* Mill.) homolog. (a) SJP39 is located in the plant cell nuclei. Subcellular localization of GFP‐SJP39 was determined in the epidermal cells of *Nicotiana benthamiana* leaves by confocal microscopy. Histone 2B (H2B)‐mCherry was used as a nuclear marker. Fluorescent signals were observed at 2 d post agroinfiltration. Bars, 50 μM. (b) Phylogenetic analysis of the VIIIb subfamily of bHLH transcription factors in *A. thaliana* and all bHLH TFs in jujube. Neighbor joining method was applied to generate the phylogenetic tree using a bootstrap value of 1000. AtbHLH87 (bold), ZjbHLH87 (bold), and the clade containing the bHLH87 proteins are highlighted. (c) Yeast two‐hybrid (Y2H) identified bHLH87 as an interactor of SJP39. Plasmids of the bait and prey pairs were co‐transformed into yeast cells and selected on double dropout (SD/−Trp/−Leu) or triple dropout (SD/−Trp/−Leu/−His) supplemented with 10 mM 3‐amino‐1,2,4‐triazole (3AT). Yeast cotransformed with AD‐AtTCP13 and BD‐SAP11 was used as a positive control. (d) SJP39 interacts with bHLH87 TFs *in vitro*. His‐AtbHLH87, His‐ZjbHLH87, glutathione S‐transferase (GST)‐SJP39, GST‐SJP39^32–77^, and GST‐SJP39^78–114^ were expressed in *Escherichia coli*. Purified protein was incubated with glutathione agarose. Coprecipitation of SJP39 with AtbHLH87 and ZjbHLH87 was detected by western blotting. SAP, *s*ecreted *A*Y‐WB *p*roteins.

To investigate how SJP39 manipulates plants, we determined its interacting proteins. Most phytoplasma effectors with known virulence targets interact with host TFs (Sugio *et al*., 2011b; Marrero *et al*., 2024), and therefore, we sought to identify plant TFs that may associate with SJP39. A Y2H screening was performed using SJP39 as the bait against a library of 1956 TFs, which represents 78.5% of the total TFs described in *A. thaliana* (Pruneda‐Paz *et al*., 2014) (Fig. S2a). This screen identified 22 TFs as potential SJP39 interactors (Table S4), which were further tested using pairwise assays. These experiments confirmed AtbHLH87 (AT3G21330) as the only TF that interacts with SJP39 (Fig. S2b). The potential interaction of SJP39 with a plant TF is also supported by the observation that SJP39 was predominantly located in the nuclei when expressed in *N. benthamiana* (Fig. 2a).

bHLH is a large TF family in *A. thaliana*; 171 bHLH TFs fall into 17 major groups (I–XVII) and 31 subfamilies (Heim *et al*., 2003). AtbHLH87 belongs to the VIIIb subfamily (Gao & Dubos, 2023). We pulled out members of the VIIIb subfamily bHLH TFs from jujube and constructed a phylogenetic tree with their *A. thaliana* homologs. This analysis revealed ZjbHLH87 (XP_015866259.1) as the closest homolog of AtbHLH87 (Figs 2b, S3). Y2H assays show that SJP39 also interacted with ZjbHLH87 (Fig. 2c).

SJP39 is a small protein that has 114 amino acids, with the N‐terminal 31 aa predicted to be the secretion SP. alphafold2 (Jumper *et al*., 2021) prediction indicates two α‐helices in the protein (Fig. S4). We made two truncates of SJP39, each containing one helix, and determined the region that is responsible for its interaction with bHLH87. Results from Y2H assays show that SJP39^32–77^ did not interact with AtbHLH87 or ZjbHLH87 (Fig. 2c). SJP39^78–114^ was self‐active in yeast; thus, we could not conclude whether this C‐terminal fragment is sufficient for bHLH87 interaction.

Protein–protein interactions were further confirmed using *in vitro* and *in planta* pull‐down assays. Purified proteins of His‐tagged bHLH87 were incubated with GST‐tagged SJP39, SJP39^32–77^, or SJP39^78–114^ and precipitated using glutathione agarose. Immunoblot analysis demonstrated that both AtbHLH87 and ZjbHLH87 could be pulled down by GST‐SJP39 but not either of the truncated proteins (Fig. 2d). Co‐immunoprecipitation assays were also conducted in *N. benthamiana* leaves co‐expressing bHLH87‐Myc and GFP‐tagged SJP39 or SJP39^32–77^. Total proteins were immunoprecipitated using anti‐GFP magnetic beads, and the coprecipitation of bHLH87 proteins was detected using anti‐Myc antibody (Fig. S5). Taken together, these experiments strongly suggest that the effector SJP39 directly interacts with the plant bHLH87 from both *A. thaliana* and jujube. Both the N‐ and C‐terminal halves of SJP39 are required for this interaction.

### bHLH87 is stabilized by SJP39

**Fig. 3:**
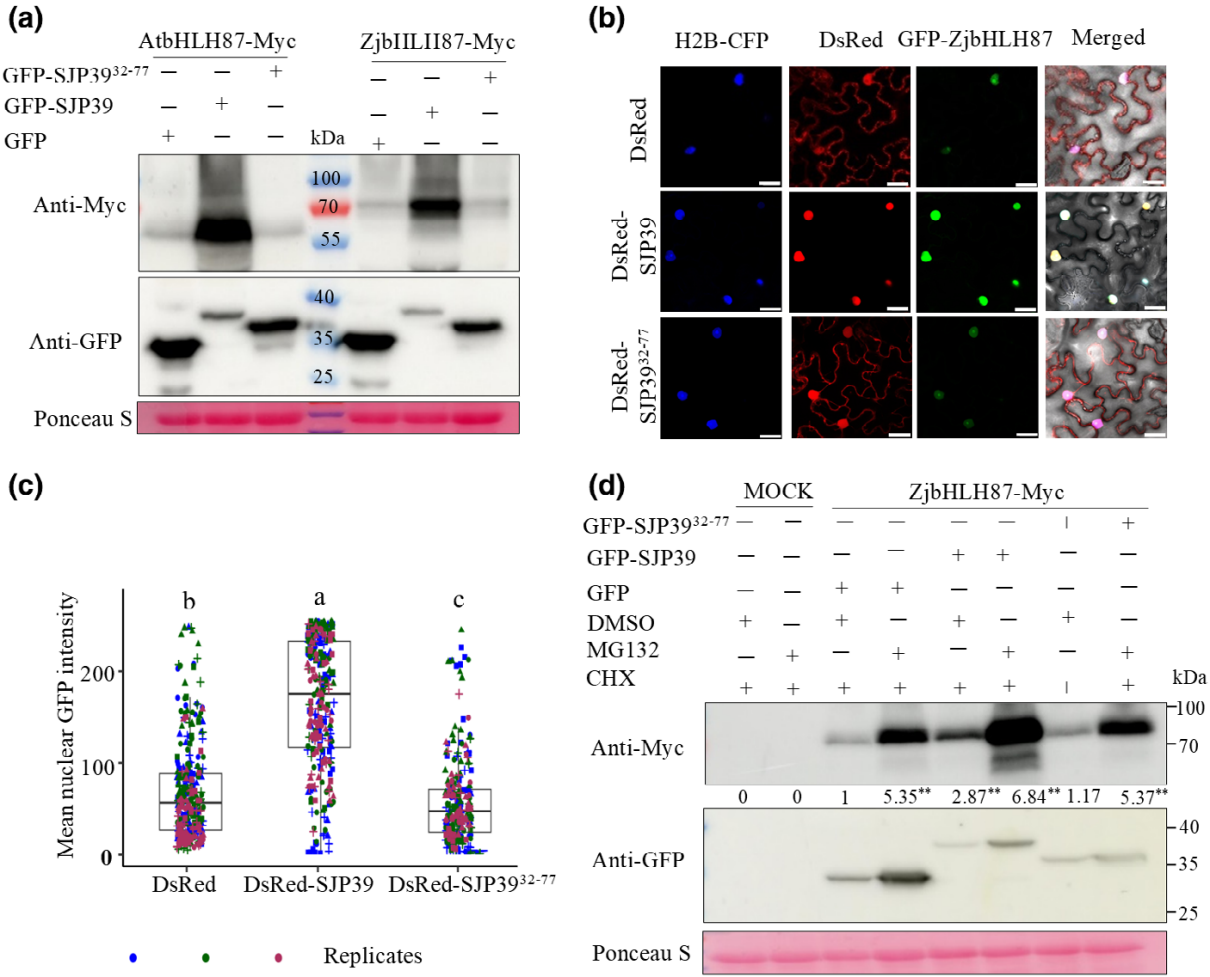
SJP39 stabilizes plant bHLH87 proteins in the nuclei. (a) SJP39 increased the accumulation of bHLH87 proteins when co‐expressed in *Nicotiana benthamiana*. Myc‐tagged AtbHLH87 and ZjbHLH87 were co‐expressed in the presence of GFP‐SJP39 or GFP‐SJP39^32–77^. Protein abundance was determined by western blotting. Ponceau S staining was used to confirm equal loading. (b) SJP39 increased the accumulation of bHLH87 proteins in the nuclei. GFP‐ZjbHLH87 was co‐expressed with DsRed‐SJP39 in *N. benthamiana*. Histone 2B (H2B)‐CFP was used as a nuclear marker. The fluorescence signals were examined using a confocal laser scanning microscope after 3 d. Bars, 25 μm. (c) Quantification of GFP fluorescence intensity in the confocal images using imagej (*n* = 80). The boxes of boxplots indicate the 25 and 75% quantiles; the horizontal line indicates the median. The whiskers extend to the largest and smallest value no further than 1.5 times the interquartile range. Different letters indicate significant differences (one‐way ANOVA, *P* < 0.05). Different colors represent three different replicates, whereas different shapes correspond to distinct plants within each replicate. (d) SJP39 stabilized bHLH87 proteins independent of 26 proteasomes. ZjbHLH87‐Myc was co‐expressed with either GFP, GFP‐SJP39, or GFP‐SJP39^32–77^ in *N. benthamiana* leaves. After 48 h, dimethyl sulfoxide (DMSO) or MG132 (100 μM) combined with cycloheximide (CHX) (100 μM) were infiltrated into the agroinfiltrated leaf areas. Six hours post treatment, total proteins were extracted and analyzed by western blotting. Ponceau S staining was used to confirm equal protein loading. Signal intensity was analyzed using imagej. The data presented are the means from three independent biological replicates, with asterisks indicating statistically significant differences compared to ZjbHLH87 levels when co‐expressed with GFP and treated with DMSO (Student's *t*‐test, **, *P* < 0.01).

Most characterized phytoplasma effectors target TFs for degradation (MacLean *et al*., 2011; Sugio *et al*., 2011a; N. Wang *et al*., 2018; Huang *et al*., 2021). We investigated whether SJP39 affected the stability of bHLH87 proteins. Co‐expression with GFP‐SJP39 in *N. benthamiana* led to a significantly increased accumulation of Myc‐bHLH87 (Fig. 3a). By contrast, bHLH87 only accumulated to a low level when co‐expressed with GFP or GFP‐SJP39^32–77^. This result is also consistent with the bHLH87 input from our Co‐IP assays (Fig. S5). To further validate this observation, we evaluated the abundance of bHLH87 in the plant cell nuclei using confocal microscopy. For this purpose, DsRed‐SJP39 or DsRed‐SJP39^32–77^ was co‐expressed with GFP‐ZjbHLH87 in *N. benthamiana* together with the nuclear marker H2B‐CFP. In general, the nuclear localization of ZjbHLH87 remained unchanged in all samples. However, in the presence of SJP39, but not SJP39^32–77^, GFP‐ZjbHLH87 exhibited significantly stronger nuclear fluorescence (Fig. 3b,c). These experiments demonstrate that SJP39 associates with plant bHLH87 in the nuclei and enhances their stability.

We next examined whether SJP39 may stabilize ZjbHLH87 by suppressing 26S proteasome‐dependent protein degradation. For this purpose, ZjbHLH87‐Myc was expressed alone or with GFP, GFP‐SJP39, or GFP‐SJP39^32–77^ in *N. benthamiana* leaves. The protein levels of ZjbHLH87 were examined after treatment with the proteasome inhibitor MG132 and CHX. We observed a significant increase in ZjbHLH87 protein levels in leaves treated with MG132 + CHX regardless of the presence or absence of SJP39, indicating that SJP39‐mediated stabilization of ZjbHLH87 is independent of the 26S proteasome (Fig. 3d).

### SJP39 induces developmental defects in *A. thaliana* and jujube

**Fig. 4:**
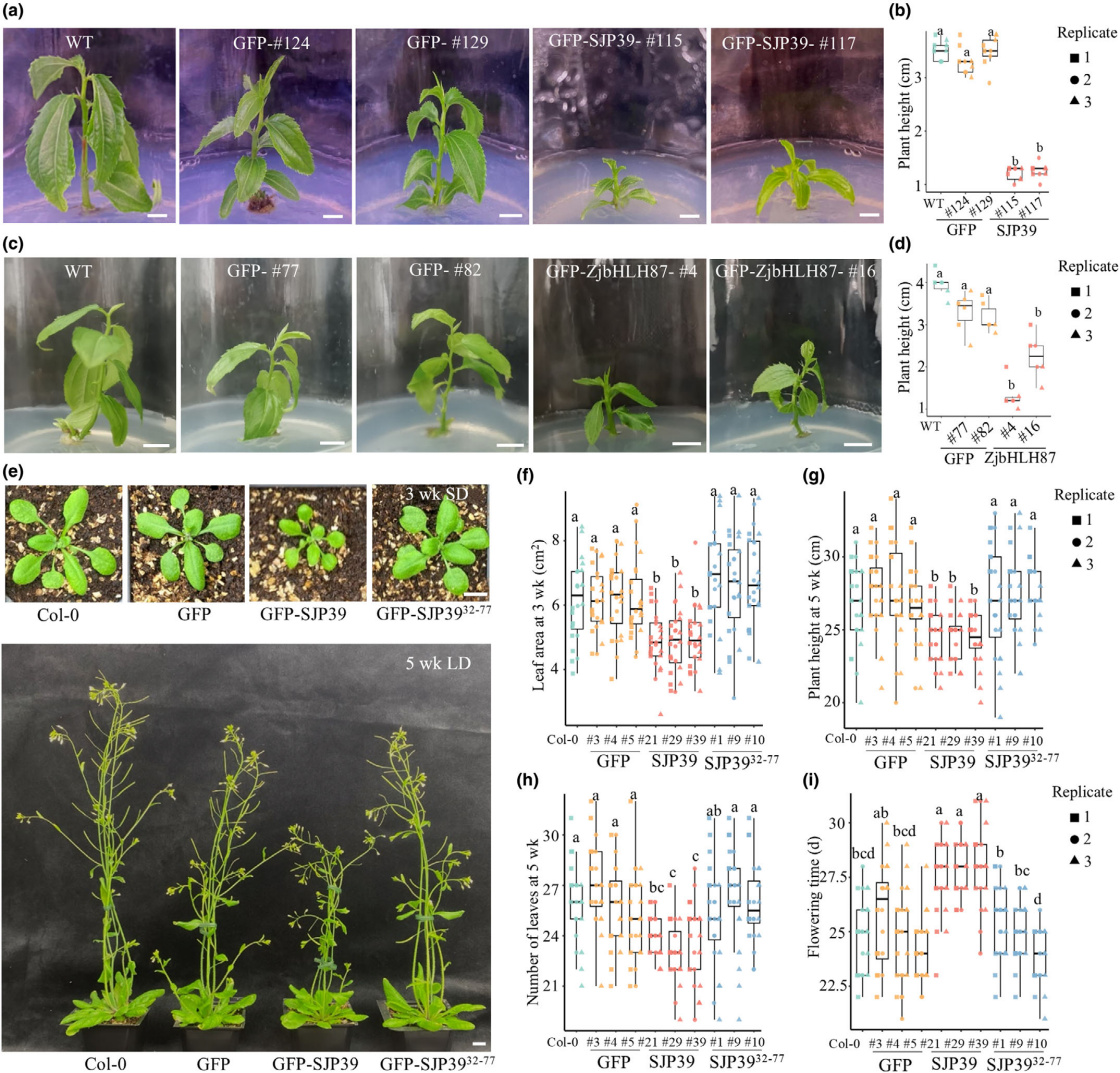
Expression of SJP39 induces developmental defects in jujube (*Ziziphus jujuba* Mill.) and *Arabidopsis thaliana*. (a, b) Jujube seedlings were stunted when expressing *SJP39*. Five‐week‐old seedlings of two independent jujube lines expressing *SJP39* or *GFP* were imaged and their height quantified (*n* = 6). Bars, 1 cm. WT, wild‐type. Different letters indicate significant differences (one‐way ANOVA analysis, *P* < 0.05). (c, d) Overexpressing *ZjbHLH87* also led to stunting of jujube seedlings (*n* = 6). Different letters indicate significant differences (one‐way ANOVA analysis, *P* < 0.05). Bars, 1 cm. (e–i) Transgenic *A. thaliana* expressing *SJP39* exhibited developmental defects. (e) Representative images of transgenic *A. thaliana* expressing *SJP39* or *GFP* at 3 wk (top) or 5 wk (bottom) after germination. Bars, 1 cm. Rosette leaf area of 3‐wk‐old plants (f), plant height (g), leaf numbers (h) of 5‐wk‐old plants, and flower time (i) were quantified (*n* = 24). Different letters label significant differences (one‐way ANOVA analysis, *P* < 0.05). In all the boxplots, the boxes indicate the 25 and 75% quantiles, with the horizontal line indicating the median and the whiskers extending to the largest/smallest value no further than 1.5 times the interquartile range.

To further characterize the role of SJP39 and the consequence of ZjbHLH87 stabilization, we generated transgenic jujube constitutively expressing either *GFP*, *GFP‐SJP39*, or *GFP‐ZjbHLH87* in WT ‘Jingzao 39’ jujube (Fig. S6a–d). Interestingly, *GFP*‐*SJP39*‐expressing seedlings exhibited smaller leaves and reduced height compared to lines expressing *GFP* or WT (Fig. 4a,b). Overexpressing *GFP‐ZjbHLH87* also led to similar phenotypes in jujube seedlings (Fig. 4c,d). Between two independent transgenic lines, line #4 exhibited more severe phenotypes compared to line #16, consistent with the higher expression level of *GFP‐ZjbHLH87* in line #4 (Fig. S6c). Jujube witches' broom infection causes short internodes, yellowing, and the development of abnormally small leaves in jujube trees (Chen *et al*., 2022; Yang *et al*., 2023). The growth defects observed in transgenic jujube resemble symptoms observed in JWB diseased trees, suggesting that SJP39 may contribute to virulence.

We also generated transgenic *A. thaliana* lines expressing *GFP*, *GFP‐SJP39*, and *GFP‐SJP39* ^*32–77*^ (Fig. S6e). Similar to what was observed in jujube, *GFP‐SJP39* transgenic lines have smaller rosette leaves (Fig. 4e,f). Additionally, 5‐wk‐old plants showed significantly reduced height and leaf number compared to Col‐0 or transgenic plants expressing *GFP* or *GFP‐SJP39* ^*32–77*^ (Fig. 4g,h). *SJP39* expression also led to delayed flowering (Fig. 4i). Similarly, transgenic *A. thaliana* lines expressing *ZjbHLH87‐Myc* (Fig. S6f) showed phenotypes resembling those of *SJP39*‐expressing lines, including reduced plant height, leaf area, and late flowering (Fig. S7). We also analyzed an *Atbhlh87* mutant of *A. thaliana*, which did not show growth defects compared to WT plants (Fig. S8). These results suggest that SJP39 impairs plant growth, mirroring disease symptoms induced by phytoplasma infection. Overexpression of *ZjbHLH87* in jujube and *A. thaliana* led to similar growth inhibition, indicating that the virulence function of SJP39 is potentially mediated by stabilizing ZjbHLH87.

### SJP39 disrupts GA signaling, potentially through interaction with ZjbHLH87

SJP39‐mediated ZjbHLH87 stabilization may alter the expression of genes within its regulon. We therefore analyzed genes regulated by SJP39 and ZjbHLH87 in jujube seedlings expressing *SJP39* or *ZjbHLH87* using RNA‐Seq. Pairwise comparisons were made between transcriptomes of *SJP39*‐ or *ZjbHLH87*‐expressing plants and seedlings transformed with *GFP*. Differentially expressed genes were defined by |log_2_ fold change (log_2_FC)| > 2 and an adjusted *P*‐value of < 0.05. *SJP39*‐ and *ZjbHLH87*‐expressing plants had 378 (Table S5) and 871 DEGs (Table S6) compared to control samples, respectively.

**Fig. 5:**
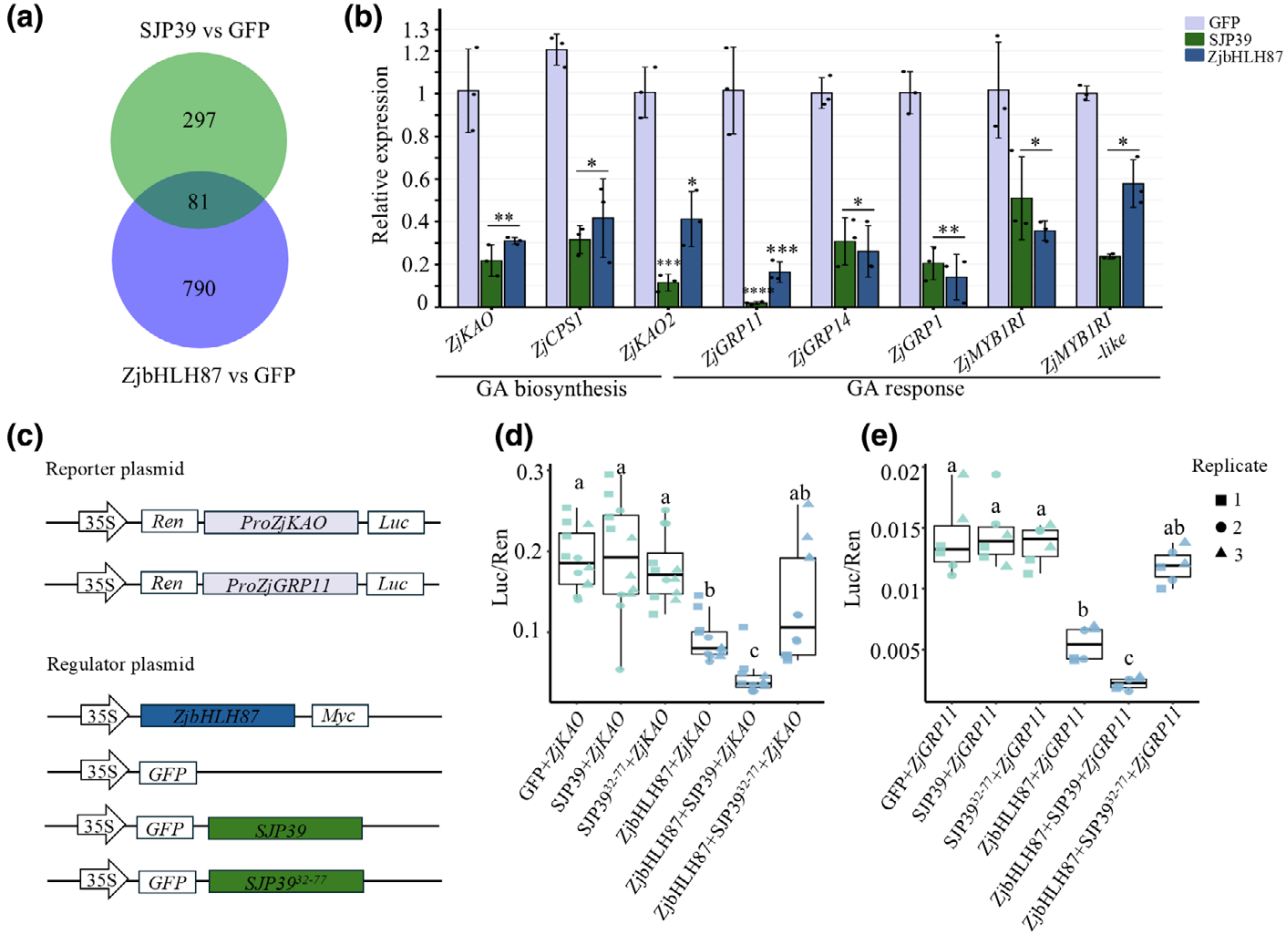
SJP39 affects gibberellin (GA) signaling in jujube (*Ziziphus jujuba* Mill.), potentially by interacting with ZjbHLH87. (a) Venn diagram of shared differentially expressed genes (DEGs) in jujube transgenic lines expressing *SJP39* or overexpressing *ZjbHLH87*. RNA‐Seq data were analyzed using pairwise comparisons between *SJP39* or *ZjbHLH87* and GFP. Differentially expressed genes were identified as genes with |log_2_ fold change (log_2_FC) > 2 and an adjusted *P*‐value of < 0.05. A full DEG list is in Supporting Information Tables S5 and S6. (b) Gibberellin‐related genes were downregulated in jujube seedling expressing *SJP39* or overexpressing *ZjbHLH87*. Relative expression of GA biosynthetic and GA‐responsive genes was determined by using RT‐qPCR. *ZjKAO: ent‐kaurenoic acid oxidase; ZjKAO2: ent‐kaurenoic acid oxidase 2; ZjCPS1: ent‐copalyl diphosphate synthase 1; ZjGRP1: GA‐regulated protein 1; ZjGRP11: GA‐regulated protein 11; ZjGRP14: GA‐regulated protein 14; ZjMYB1R1: transcription factor MYB1R1; ZjMYB1R1‐like: transcription factor MYB1R1‐like. ZjACT1* was used as an internal control. Data are means ± SD (*n* = 3). Asterisks indicate significantly different expression *, *P* < 0.05; **, *P* < 0.01; ***, *P* < 0.001; ****, *P* < 0.001 (Student's *t*‐test). (c–e) SJP39 enhanced the regulation of GA‐related genes by ZjbHLH87. Constructs used in the dual‐luciferase (LUC) reporter assay (c). The CaMV35S promoter was used to drive the expression of Renilla (REN) LUC, whereas the LUC was cloned after the promoter of *ZjKAO* or *ZjGRP11*. The constructs were introduced into *Agrobacterium*, which were used for transient expression in *Nicotiana benthamiana*. LUC/REN ratios were used to indicate regulation of the *ZjKAO* promoter (d) or *ZjGRP11* promoter (e) by ZjbHLH87 in the presence of absence of SJP39 (*n* = 12). The boxes of boxplots indicate the 25 and 75% quantiles, and the horizontal line indicates the median. The whiskers extend to the largest and smallest value no further than 1.5 times the interquartile range. Different letters label significant differences (one‐way ANOVA analysis, *P* < 0.05).

Importantly, 81 DEGs were shared between SJP39 and ZjbHLH87 (Table S7), indicating that they could trigger similar alterations in gene expression (Fig. 5a). GO analysis of these shared DEGs showed enriched cellular processes associated with anatomical structure (phloem) development, developmental processes, diterpenoid metabolism, and lipid metabolism, which were downregulated, while cytokinin signaling was upregulated (Fig. S9).

We were intrigued by the downregulation of the diterpenoid metabolic process in *SJP39*‐ and *ZjbHLH87*‐expressing plants. Most DEGs within the diterpenoid metabolic pathway were downregulated (Fig. S9). Interestingly, AtbHLH87 was reported to interact with the DELLA protein GA insensitive, which is a negative regulator of GA signaling (de la Marín‐ Rosa *et al*., 2014). Gibberellins are plant hormones that are regulated by the diterpenoid pathway. DELLA proteins are key regulators of GA signaling. Further analysis focusing on genes involved in GA biosynthesis and response pathways revealed several genes that were downregulated in both *SJP39*‐ and *ZjbHLH87*‐expressing transgenic lines (Figs S10, S11). These include genes encoding ent‐KAO and GA‐regulated protein 11 (GRP11), which are known to play crucial roles in GA biosynthesis and response, respectively (Nahirñak *et al*., 2012; Regnault *et al*., 2014). This trend of differential expression in GA‐related genes was confirmed using reverse transcription‐quantitative polymerase chain reaction (Fig. 5b). In addition to GA‐related pathways, we also observed GO term enrichment of DEGs associated with defense‐related processes, such as stomatal movement, reactive oxygen species (ROS) production/detoxification, as well as JA and cytokinin pathways (Tables S8, S9; Fig. S12).

The transcriptomic analysis indicates a potential relationship between SJP39/ZjbHLH87‐mediated regulation of plant growth and the GA pathway. We further examined the direct regulation of ZjbHLH87 on the GA‐related gene expression as well as the impact of SJP39 on the regulatory activity of ZjbHLH87 using dual‐LUC assays. Co‐expression of ZjbHLH87 significantly decreased the LUC reporter gene expression driven by the promoter of *ZjKAO* or *ZjGRP11* in *N. benthamiana* (Figs 5c–e, S13). Furthermore, co‐expression with SJP39 resulted in a further decrease in ZjbHLH87‐mediated transcription suppression, while this effect was not observed in the presence of SJP39^32–77^ (Fig. 5d,e). These findings indicate that ZjbHLH87 negatively regulates the expression of *ZjKAO* and *ZjGRP11*, which is enhanced by SJP39 stabilization.

**Fig. 6:**
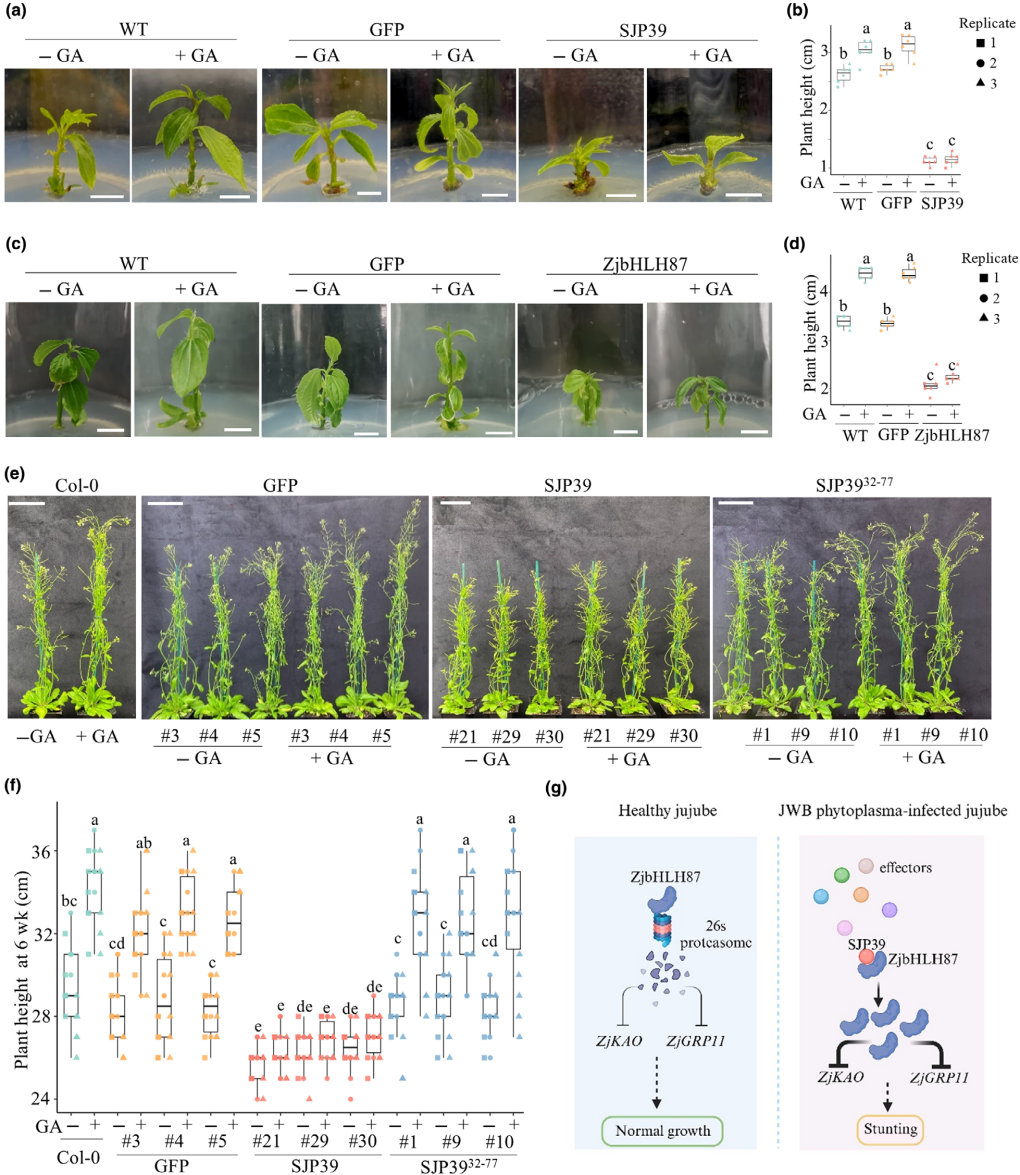
Jujube (*Ziziphus jujuba* Mill.) and *Arabidopsis thaliana* expressing SJP39 are insensitive to gibberellin (GA) treatment. (a, c) Images of wild‐type (WT) 5‐wk‐old jujube seedlings or jujube expressing GFP, SJP39, or ZjbHLH87 with (+) or without (−) 2 mg l^−1^ GA treatment. Bars, 1 cm. (b, d) Height of the jujube seedlings (*n* = 6). The boxes of boxplots indicate the 25 and 75% quantiles; the horizontal line indicates the median. The whiskers extend to the largest and smallest value no further than 1.5 times the interquartile range. (e) Images of 6‐wk‐old Col‐0 or *A. thaliana* expressing GFP, SJP39 orSJP3932‐77 with (+) or without (−) 100 μM GA treatment. Bars, 5 cm. (f) Height of 6‐wk‐old *A. thaliana* plants (*n* = 18). Significant differences are indicated by different letters (one‐way ANOVA, *P* < 0.05). (g) A proposed model illustrating how SJP39 may affect the growth of jujube by stabilizing the host transcription factor bHLH87. In jujube plants infected with jujube witches' broom (JWB) phytoplasma, SJP39 interacts with ZjbHLH87 in the nuclei, and this interaction leads to increased accumulation of ZjbHLH87 in a 26S proteasome‐independent manner. ZjbHLH87 acts as a negative regulator of GA‐related genes such as *ZjKAO* and *ZjGRP11*. SJP39‐mediated stabilization of ZjbHLH87 results in dwarfism, mimicking disease symptoms observed in JWB‐infected jujube. This figure was created in BioRender (Lab, 2025; https://BioRender.com/a74w586). The solid arrows and blunt‐ended bars represent positive and negative regulation, respectively. Dashed arrows indicate predicted pathways.

To further investigate the relationship between SJP39, ZjbHLH87, and GA, we subjected *SJP39* and *ZjbHLH87* transgenic jujube lines to GA treatment. Gibberellin application significantly induced growth in WT and *GFP* lines (Fig. 6a–d). Gibberellin application was also performed on *GFP*, *SJP39*, and *SJP39* ^*32–77*^ transgenic *A. thaliana* lines (Fig. 6e). Consistent with observations in jujube, *SJP39* transgenic lines showed impaired GA responses compared to Col‐0 or transgenic plants expressing *GFP* or *SJP39* ^*32–77*^ (Fig. 6f). These results provide further evidence that the expression of *SJP39* or overexpression of *ZjbHLH87* may lead to developmental defects associated with phytoplasma infection in jujube by inhibiting gene expression in the GA pathway.

## Discussion

Jujube is a valuable perennial fruit crop belonging to the Rhamnaceae family, with over 900 known cultivars (Zhao *et al*., 2019). Many of the jujube cultivars are susceptible to JWB, a severe and often fatal disease (Liu & Zhao, 2009). Although jujube germplasm with various levels of tolerance/resistance to JWB has been identified (Song *et al*., 2019), the underlying mechanism remains unknown. A deeper understanding of the molecular interactions between phytoplasma and jujube plants that are associated with pathogenesis is required for effective disease management.

In our investigation of JWB disease, we first conducted a genome‐wide analysis of putative effector proteins, which revealed 72 candidates. This number is significantly higher than the 43 effectors reported in a previous publication (Deng *et al*., 2021). This difference could be attributed to our use of signalp v.3.0, v.4.0, and v.5.0 for effector prediction, as opposed to signalp v.4.0 alone. Indeed, it was reported that relying solely on signalp v.4.0 may result in an underestimation of candidate effectors (Chen *et al*., 2022). Among the 72 candidates, we focused on SJP39, one of the most highly expressed effectors in JWB phytoplasma‐infected jujube trees, particularly in the shoots. Notably, SJP39 homologs are produced by several other phytoplasmas that infect a broad range of plants, suggesting a conserved role in promoting phytoplasma infection.

Interaction between pathogen effectors and host TFs is a commonly used mechanism by which pathogens modulate host physiological processes (Tsuda & Somssich, 2015). Phytoplasma effectors predominantly target host TFs, and in most cases, degrade these TFs to interrupt their regulatory functions (MacLean *et al*., 2011; Sugio *et al*., 2011a; Huang *et al*., 2021; Chen *et al*., 2022). SJP39 is nearly exclusively located in the nuclei when expressed in *N. benthamiana*, consistent with its interaction with the bHLH family TF, bHLH87, as a host target. Unlike most phytoplasma effectors, SJP39 was found to stabilize bHLH87. In the absence of SJP39, ZjbHLH87 undergoes rapid degradation via the 26S proteasome, a common fate for TFs in response to hormonal or environmental cues (Jia *et al*., 2023). SJP39 stabilizes ZjbHLH87 independent of the 26S proteasome pathway. Future research understanding the underlying mechanism will provide further information on the virulence activity of SJP39. While our study identified ZjbHLH87 as a key target of SJP39 in jujube, it is important to acknowledge that our screen was primarily based on an *A. thaliana* library that covers 78.4% of its known TFs. Therefore, we may not fully capture plant TFs that could be targeted by SJP39. Moreover, SJP39 may target lineage‐specific TFs in *Z. jujuba* in addition to ZjbHLH87.

bHLH TFs have been found to be targeted by effectors of other pathogens (Schwartz *et al*., 2017; Turnbull *et al*., 2017). The bacterial pathogen *Xanthomonas gardneri* produces an effector AvrHah1, which activates bHLH3 and bHLH6 TFs in tomato plants. These bHLH TFs regulate genes involved in pectin modification, enhancing water uptake and tissue damage, thus promoting bacterial spread (Schwartz *et al*., 2017). The oomycete pathogen *Phytophthora infestans* produces an effector AVR2, which induces the expression of brassinosteroid (BR)‐responsive genes in potato, including the bHLH TF StCHL1. Silencing *StCHL1* reduced pathogen colonization, indicating that this TF is a positive regulator of plant immunity (Turnbull *et al*., 2017). Therefore, bHLH TFs with diverse functions are exploited by different pathogens to facilitate their infection.

Typical symptoms caused by phytoplasma infection include severe developmental abnormalities, such as increased lateral branching and leafy flowers. The JWB phytoplasma effectors, SJP1, SJP2, and SJP3, induced phenotypes related to these symptoms when expressed in *N. benthamiana* (Zhou *et al*., 2021; Deng *et al*., 2024). In addition, infected jujube trees often exhibit stunted growth. Interestingly, jujube seedlings expressing *SJP39* were significantly smaller than controls. Similarly, overexpression of *ZjbHLH87* also led to stunted growth in jujube, consistent with the possibility that the growth defects caused by SJP39 can be attributed to its stabilization of ZjbHLH87. While we do not have data on transgenic jujube, transgenic *A. thaliana* expressing SJP39 also showed delayed flowering, which is another symptom observed in phytoplasma‐infected trees. As such, SJP39 may contribute to the disease symptom development after phytoplasma infection of jujube trees.

Transcriptome analysis indicates a potential disruption in the GA pathway by SJP39. We found ZjbHLH87 can directly regulate the expression of *ZjKAO* and *ZjGRP11*. Kaurenoic acid oxidase belongs to the CYP88A subfamily of cytochrome P450 monooxygenases, converting ent‐KA to GA12, the precursor of all GAs (Huang *et al*., 2012). *Arabidopsis thaliana* has two KAO‐encoding genes, *KAO1* and *KAO2*. The *kao1 kao2* double mutant exhibits severe GA‐deficient phenotypes, including impaired seed germination and dwarfism (Regnault *et al*., 2014). Degradation of DELLA proteins in the presence of GAs promotes the activation of various transcriptional regulators, including gibberellic acid‐stimulated *Arabidopsis* (GASA), a GA‐responsive protein that contributes to plant growth and development (Bouteraa *et al*., 2023). In *A. thaliana*, GASA14 enhances plant growth through GA‐induced and DELLA‐dependent signaling (Sun *et al*., 2013). We found that the jujube homologs of *GASA*, called *ZjGRPs*, were downregulated in transgenic jujube lines expressing *SJP39* or *ZjbHLH87*. Therefore, SJP39‐induced suppression of GA signaling is likely mediated by ZjbHLH87. This is consistent with the observation that transgenic jujube plants expressing *SJP39* or overexpressing *ZjbHLH87* were largely unresponsive to exogenous GA treatment.

Phytoplasmas specifically colonize the phloem tissue of host plants. Gibberellins have been reported to modulate phloem sieve tube differentiation through callose deposition at sieve plates (An *et al*., 2020). SJP39‐mediated disruption of GA signaling could thus impair phloem functions, such as sugar transport dynamics, which are observed in JWB‐infected jujube (Liu *et al*., 2021). This manipulation may also explain the symptoms of JWB‐diseased plants as impaired phloem function can lead to systemic metabolic imbalances and growth abnormalities.

Modulation of the host GA pathway is a common strategy utilized by both pathogenic and mutualistic microbes. For instance, the rice blast fungus *Magnaporthe oryzae* elevates GA signaling to enhance host susceptibility, although the underlying molecular mechanisms remain unclear (Yang *et al*., 2008). Conversely, the arbuscular mycorrhizal fungus *Rhizophagus irregularis* suppresses GA signaling through the GAI‐RGA‐ and ‐SCR (GRAS) TF Mig1, which interacts with DELLA proteins and promotes arbuscule development (Heck *et al*., 2016). The interaction of SJP39 with bHLH87 provides a novel mechanism through which plant‐associating microbes can manipulate the GA pathway in their plant hosts to facilitate interaction.

There is a complex interplay between GA and other plant hormones. For instance, GA‐triggered degradation of DELLA proteins suppresses JA signaling (Hou *et al*., 2010). DELLA also regulates immune responses by modulating the balance of JA and salicylic acid (SA) signaling (Navarro *et al*., 2008). In addition, cytokinin can regulate SA (Choi *et al*., 2011) and GA biosynthesis (Zubo & Schaller, 2020). Indeed, transgenic jujube plants expressing SJP39 or bHLH87 also exhibited DEG enrichment for GO terms associated with JA and cytokinin pathways. Therefore, SJP39 may directly or indirectly regulate the phytohormone network to facilitate infection. Further analysis of relevant hormone levels in SJP39‐expressing transgenic jujube plants as well as in JWB‐diseased trees will help dissect this process.

Overall, our study established a role of an important JWB phytoplasma effector, SJP39, which interacts with and stabilizes the jujube TF ZjbHLH87 (Fig. 6g). This misregulation of the ZjbHLH87 regulon results in developmental abnormalities, especially stunted growth and potentially delayed flowering, which are relevant to the disease symptoms of JWB. This finding significantly advances our understanding of the molecular mechanisms by which JWB phytoplasma causes disease in jujube and can be leveraged to develop management strategies.

## Competing interests

None declared.

## Author contributions

WM and XP conceived the project and guided the execution of the experiments. SY and AHL performed the experiments and analyzed the data. YY and YG contributed to generating transgenic jujube lines. HN provided assistance to the VIVE assay and yeast two‐hybrid (Y2H) screening. WC and WB supported the RNA‐Seq analysis. SY and AHL prepared figures and tables. DHN guided the Y2H screening. WM, SY and AHL wrote the manuscript with contributions from all authors.

## Disclaimer

The New Phytologist Foundation remains neutral with regard to jurisdictional claims in maps and in any institutional affiliations.

## Supporting information


**Fig. S1** Confirmation of SJP39 and SJP37 expression in *Nicotiana benthamiana*.
**Fig. S2** Yeast two‐hybrid (Y2H) screening reveals that SJP39 interacts with AtbHLH87.
**Fig. S3** Phylogenetic analysis of bHLH transcription factors in jujube.
**Fig. S4** Structure prediction of SJP39 and the truncated constructs SJP39^32–77^ and SJP39^78–114^.
**Fig. S5** SJP39 interacts with bHLH87 using the co‐immunoprecipitation assay.
**Fig. S6** Confirmation of SJP39 and ZjbHLH87 expression in transgenic plants.
**Fig. S7** Expression of *ZjbHLH87* in *Arabidopsis thaliana‐*induced developmental defects.
**Fig. S8** *Atbhlh87* mutant *Arabidopsis thaliana* did not show growth defects.
**Fig. S9** Significantly enriched Gene Ontology (GO) terms in differentially expressed genes shared in transgenic jujube expressing *SJP39* and *ZjbHLH87*.
**Fig. S10** Expression patterns of gibberellin (GA) pathway genes in *SJP39* and *ZjbHLH87* transgenic jujube lines.
**Fig. S11** Expression changes of genes involved in gibberellin (GA) biosynthesis and response pathways in *SJP39* and *ZjbHLH87* transgenic jujube.
**Fig. S12** Expression patterns of jasmonic acid (JA) pathway genes in *SJP39* and *ZjbHLH87* transgenic jujube lines.
**Fig. S13** Western blots confirming the expression of ZjbHLH87 and SJP39 in the dual‐luciferase (LUC) assay.

**Table S1** Predicted effector candidates from jujube witches' broom (JWB) phytoplasma.
**Table S2** DNA constructs used in this study.
**Table S3** Primers used in this study.
**Table S4** SJP39‐associating transcription factors detected by yeast‐two‐hybrid screening.
**Table S5** Genes differentially expressed in transgenic jujube (*Ziziphus jujuba* Mill.) plants expressing *SJP39* vs *GFP*.
**Table S6** Genes differentially expressed in transgenic jujube (*Ziziphus jujuba* Mill.) plants expressing *ZjbHLH87* vs *GFP*.
**Table S7** Eighty‐one genes differentially expressed in jujube (*Ziziphus jujuba* Mill.) transgenic lines expressing *SJP39* or *ZjbHLH87* compared to *GFP*.
**Table S8** Significant Gene Ontology terms enriched in 378 genes differentially expressed between jujube (*Ziziphus jujuba* Mill.) transgenic lines expressing *SJP39* vs *GFP*.
**Table S9** Significant Gene Ontology terms enriched in 871 genes differentially expressed between jujube (*Ziziphus jujuba* Mill.) transgenic lines expressing *ZjbHLH87* vs *GFP*.
Please note: Wiley is not responsible for the content or functionality of any Supporting Information supplied by the authors. Any queries (other than missing material) should be directed to the *New Phytologist* Central Office.

## Data Availability

The data that support the findings of this study are available within the article and in the Supporting Information (Figs S1–S13 and Tables S1–S9). The phytoplasma RNA‐Seq data have been deposited into the NCBI database under the accession no. PRJNA1158699. The transgenic jujube RNA‐Seq data have been deposited into the ENA database (BioProject No. PRJEB81825).
