# Supplementary material for: A witches' broom phytoplasma effector induces stunting by stabilizing a bHLH transcription factor in Ziziphus jujuba plants
Source: New Phytol. 2025 May 9;247(1):249–64. doi: 10.1111/nph.70172 (PMC12138163; doi:10.1111/nph.70172)
Supplement: Supplementary file 1 — Fig. S1 Confirmation of SJP39 and SJP37 expression in Nicotiana benthamiana. Fig. S2 Yeast two‐hybrid (Y2H) screening reveals that SJP39 interacts with AtbHLH87. Fig. S3 Phylogenetic analysis of bHLH transcription factors in jujube. Fig. S4 Structure prediction of SJP39 and the truncated constructs SJP3932–77 and SJP3978–114. Fig. S5 SJP39 interacts with bHLH87 using the co‐immunoprecipitation assay. Fig. S6 Confirmation of SJP39 and ZjbHLH87 expression in transgenic plants. Fig. S7 Expression of ZjbHLH87 in Arabidopsis thaliana‐induced developmental defects. Fig. S8 Atbhlh87 mutant Arabidopsis thaliana did not show growth defects. Fig. S9 Significantly enriched Gene Ontology (GO) terms in differentially expressed genes shared in transgenic jujube expressing SJP39 and ZjbHLH87. Fig. S10 Expression patterns of gibberellin (GA) pathway genes in SJP39 and ZjbHLH87 transgenic jujube lines. Fig. S11 Expression changes of genes involved in gibberellin (GA) biosynthesis and response pathways in SJP39 and ZjbHLH87 transgenic jujube. Fig. S12 Expression patterns of jasmonic acid (JA) pathway genes in SJP39 and ZjbHLH87 transgenic jujube lines. Fig. S13 Western blots confirming the expression of ZjbHLH87 and SJP39 in the dual‐luciferase (LUC) assay. [file NPH-247-249-s001.pdf]

*New Phytologist* **Supporting Information**

Article title: **A Witches' Broom Phytoplasma effector induces stunting by stabilizing a bHLH transcription factor in *Ziziphus jujuba* plants**

Authors: Shuang Yang, Amelia H Lovelace, Yi Yuan, Haizhen Nie, Weikai Chen, Yi Gao, Wenhao Bo, Dawn H Nagel, Xiaoming Pang, Wenbo Ma

Article acceptance date: 3 April 2025

The following Supporting Information is available for this article:

**Figure S1.** Confirmation of SJP39 and SJP37 expression in *Nicotiana benthamiana*.

**Figure S2.** Yeast two-hybrid (Y2H) screening reveals that SJP39 interacts with AtbHLH87.

**Figure S3.** Phylogenetic analysis of bHLH transcription factors in jujube.

**Figure S4.** Structure prediction of SJP39 and the truncated constructs $SJP39^{32\text{-}77}$ and $SJP39^{78\text{-}114}$.

**Figure S5.** SJP39 interacts with bHLH87 using co-immunoprecipitation assay.

**Figure S6.** Confirmation of SJP39 and ZjbHLH87 expression in transgenic plants.

**Figure S7.** Expression of *ZjbHLH87* in *A. thaliana* induced developmental defects.

**Figure S8.** *Atbhlh87* mutant *A. thaliana* did not show growth defects.

**Figure S9.** Significantly enriched Gene Ontology (GO) terms in DEGs shared in transgenic jujube expressing *SJP39* and *ZjbHLH87*.

**Figure S10.** Expression patterns of gibberellin (GA) pathway genes in *SJP39* and *ZjbHLH87* transgenic jujube lines.

**Figure S11.** Expression changes of genes involved in Gibberellin (GA) biosynthesis and response pathways in *SJP39* and *ZjbHLH87* transgenic jujube.

**Figure S12.** Expression patterns of jasmonic acid (JA) pathway genes in *SJP39* and *ZjbHLH87* transgenic jujube lines.

**Figure S13.** Western blots confirming the expression of ZjbHLH and SJP39 in the dual-luciferase

(LUC) assay.

**Table S1.** Predicted effector candidates from jujube witches' broom (JWB) phytoplasma.

**Table S2.** DNA constructs used in this study.

**Table S3.** Primers used in this study.

**Table S4.** SJP39-associating transcription factors detected by Yeast-two-hybrid screening.

**Table S5.** Genes differentially expressed in transgenic jujube (*Ziziphus jujuba* Mill.) plants expressing *SJP39* vs *GFP*.

**Table S6.** Genes differentially expressed in transgenic jujube (*Ziziphus jujuba* Mill.) plants expressing *ZjbHLH87* vs *GFP*.

**Table S7.** 81 genes differentially expressed in jujube (*Ziziphus jujuba* Mill.) transgenic lines expressing *SJP39* or *ZjbHLH87* compared to *GFP*.

**Table S8.** Significant GO terms enriched in 378 genes differentially expressed between jujube (*Ziziphus jujuba* Mill.) transgenic lines expressing *SJP39* vs *GFP*.

**Table S9.** Significant GO terms enriched in 871 genes differentially expressed between jujube (*Ziziphus jujuba* Mill.) transgenic lines expressing *ZjbHLH87* vs *GFP*.

**Figure S1.** Confirmation of SJP37 and SJP39 expression in *Nicotiana benthamiana*.

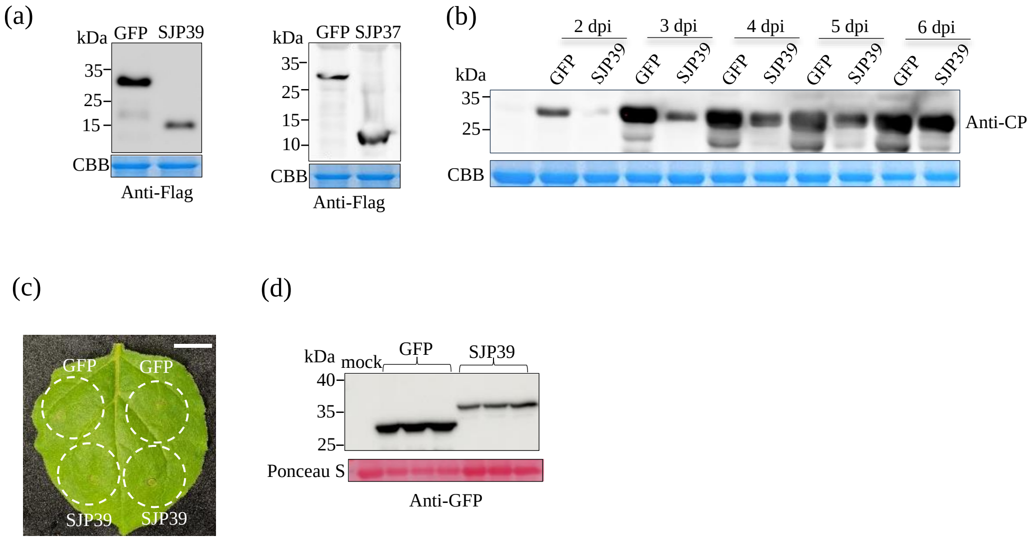


**(a)** Western blots confirming the expression of GFP-3×Flag, SJP39-3×Flag and SJP37-3×Flag protein in *N. benthamiana* using an anti-Flag antibody. Coomassie Brilliant Blue (CBB) staining was used as a loading control. **(b)** Western blot detection of PVX coat protein (CP) in *N. benthamiana* infiltrated with *Agrobacterium* carrying PVX-GFP or PVX-SJP39 using an anti-CP antibody. dpi = days post inoculation (dpi). Equal loading was confirmed by Coomassie Brilliant Blue (CBB) staining. **(c)** SJP39 expression in *N. benthamiana* did not cause visible phenotype. GFP or GFP-SJP39 were transiently expressed in *N. benthamiana*. The image was taken at 5 days post agroinfiltration (n=8). Scale bar = 1 cm. **(d)** Western blot detection of GFP and GFP-SJP39 protein expression using an anti-GFP antibody, Ponceau S staining was used to confirm equal protein loading.

**Figure S2.** Yeast two-hybrid (Y2H) screening reveals the interaction of SJP39 with the transcription factor AtbHLH87.

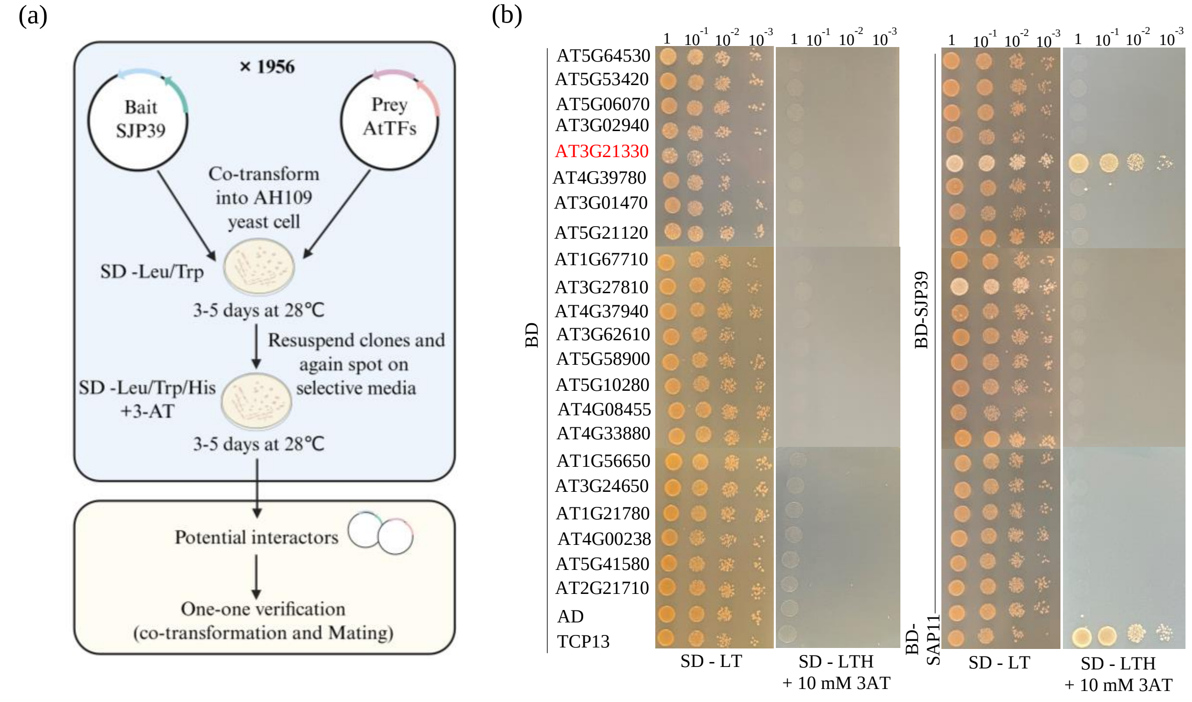


**(a)** A diagram showing the Y2H screening procedure. The blue-coloured region in the bait vector represents the GAL4 activation domain (AD) and the green-coloured region represents SJP39. The purple-coloured region in the prey vector denotes the GAL4 DNA binding domain (BD) and the pink-coloured region represents individual *A. thaliana* transcription factors. This figure was created in BioRender (Lab, W. (2025) https://BioRender.com/vdv0160). **(b)** AtbHLH87 (AT3G21330) was identified as an interactor of SJP39. Plasmids of the bait and prey pairs were co-transformed into yeast cells and selected on double dropout (SD/-Trp/-Leu) or triple dropout (SD/-Trp/-Leu/-His) supplemented with 10 mM 3-amino-1,2,4-triazole (3-AT). Yeast co-transformed with AD-AtTCP13 and BD-SAP11 was used as a positive control.

**Figure S3.** Phylogenetic analysis of bHLH transcription factors in jujube.

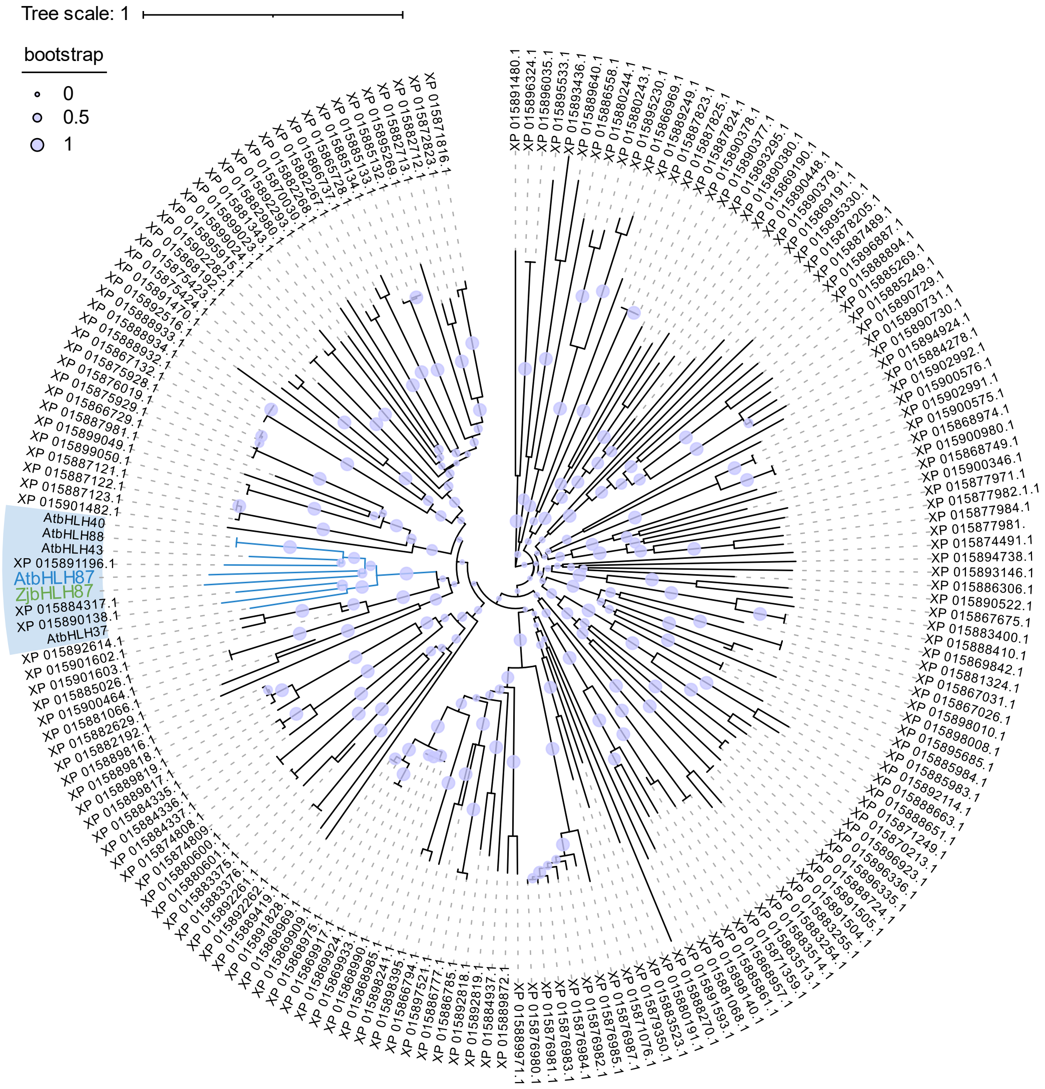


Neighbour joining method was applied to generate the phylogenetic tree using a bootstrap value of 1000. Members of the VIIIb subfamily of bHLH transcription factors in *A. thaliana* and all bHLH transcription factors encoded in the jujube genome were included in this analysis. AtbHLH87 (blue), ZjbHLH87 (green) and the clade containing the bHLH87 proteins are highlighted.

**Figure S4.** Structure prediction of SJP39 and the truncated constructs SJP39$^{32\text{-}77}$ and SJP39$^{78\text{-}114}$.

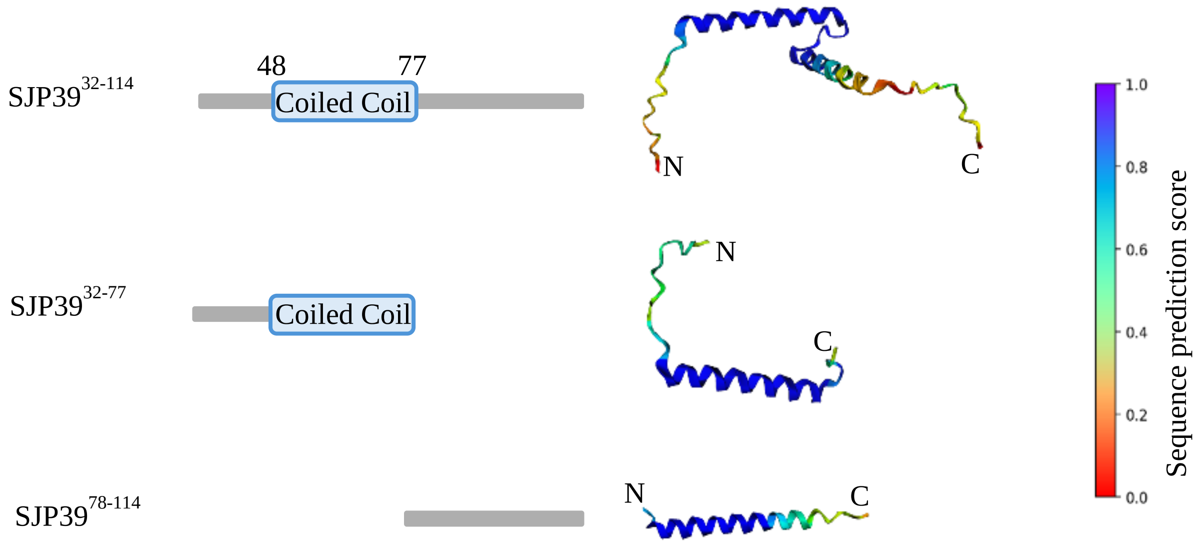

**Figure S5.** SJP39 interacts with bHLH87 using co-immunoprecipitation assay.

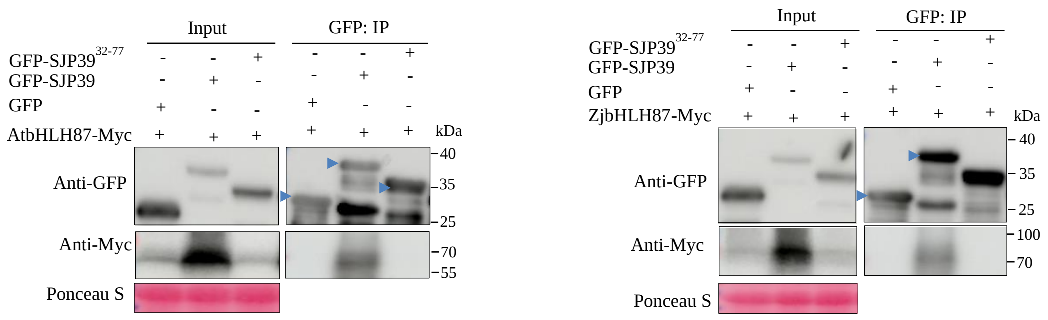


AtbHLH87-Myc, ZjbHLH87-Myc were co-expressed with GFP-SJP39 or GFP-SJP39$^{32\text{-}77}$ in *N. benthamiana*. The immune complexes were immobilized on anti-GFP magnetic beads, and the co-precipitation of AtbHLH87 and ZjbHLH87 was examined by western blotting using an anti-Myc antibody. Ponceau S staining was used to confirm equal protein loading. The blue arrowheads indicate the target bands.

**Figure S6.** Confirmation of SJP39 and ZjbHLH87 expression in transgenic plants.

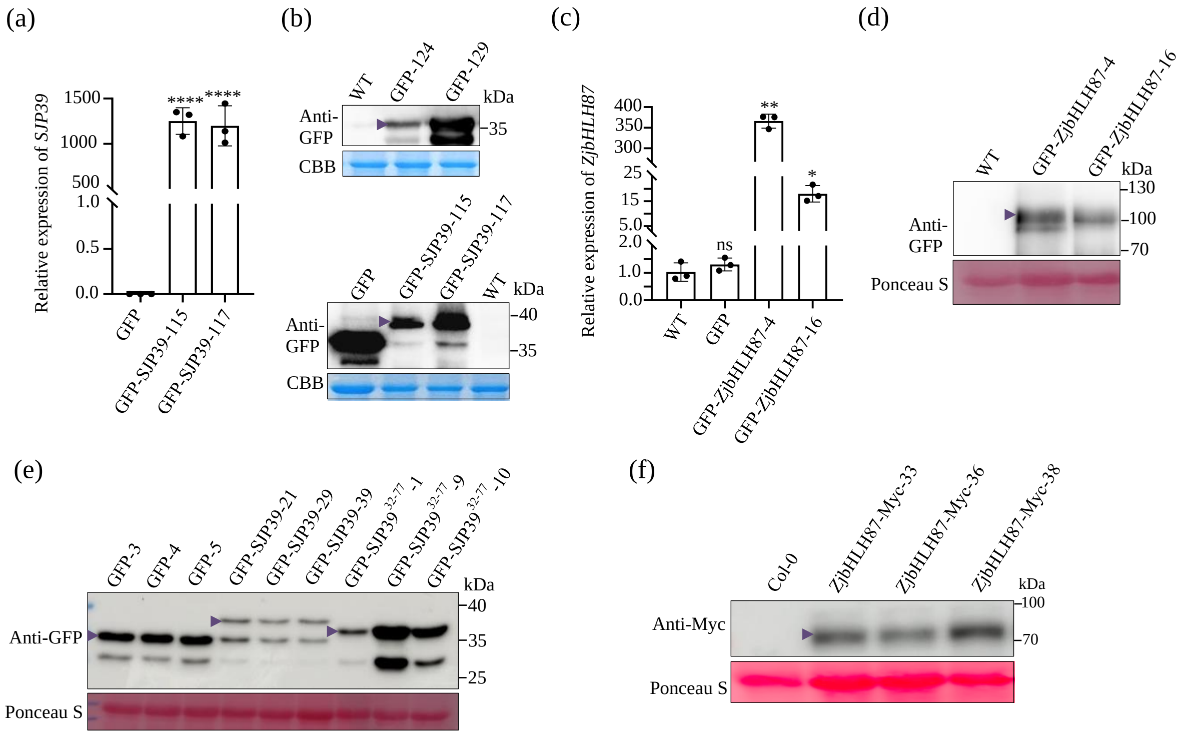


**(a, c)** RT-qPCR analysis of *SJP39* and *ZjbHLH87* transcripts in transgenic jujube. Transcript levels of *SJP39* and *ZjbHLH87* were normalized using *ZjACT1* as an endogenous control. Data are means ± SD (n = 3). Asterisks indicate significant differences (Student's t-test, *p < 0.05, **p < 0.01, ***p < 0.001, ****p < 0.0001, ns = not significant). **(b, d)** Western blot analysis of GFP, GFP-SJP39 and GFP-ZjbHLH87 proteins in transgenic jujube using an anti-GFP antibody. Coomassie Brilliant Blue (CBB) staining or Ponceau S staining were used as a loading control. The blue arrowheads indicate the target bands. **(e, f)** Western blot analysis of GFP, GFP-SJP39 and GFP-SJP39$^{32\text{-}77}$ using an anti-GFP antibody or ZjbHLH87 proteins using an anti-Myc antibody in transgenic *A. thaliana* lines. Ponceau S staining was used to confirm equal protein loading. The blue arrowheads indicate the target bands.

**Figure S7.** Expression of *ZjbHLH87* in *A. thaliana* induced developmental defects.

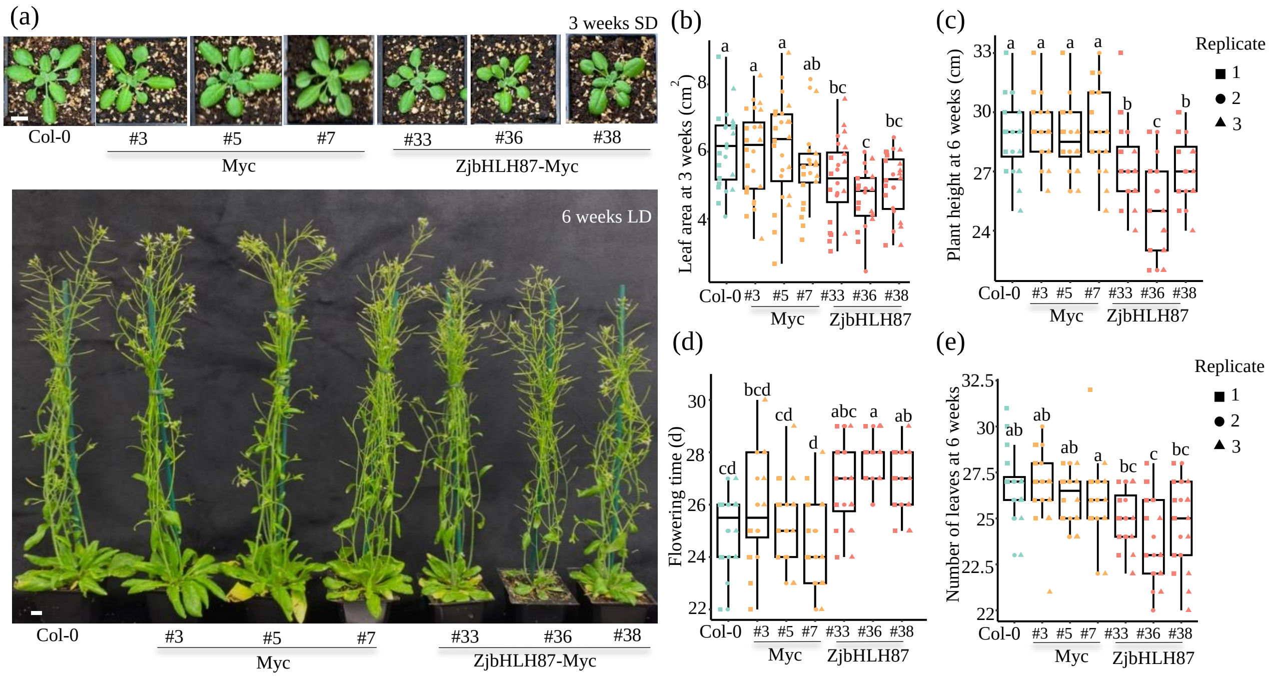


**(a)** Representative images of transgenic plants expressing ZjbHLH87-Myc or Myc (control) compared to wildtype Col-0. Images were taken at 3 weeks under short-day (SD) condition (top) or 6 weeks under long-day (LD) condition (bottom). Scale bars = 1 cm. (**b**) 3-week-old plants were analysed for rosette leaf area. **(c-e)** 6-week-old plants were analysed for plant height **(c)**, flowering time **(d)**, and leaf numbers **(e)**. The boxes of boxplots indicate the 25% and 75% quantiles, and the horizontal line indicates the median. The whiskers extend to the largest/smallest value no further than 1.5 times the interquartile range (n = 24). Different letters label significant differences using one-way ANOVA analysis (n=8, $p < 0.05$).

**Figure S8**. *Atbhlh87* mutant *A. thaliana* did not show growth defects.

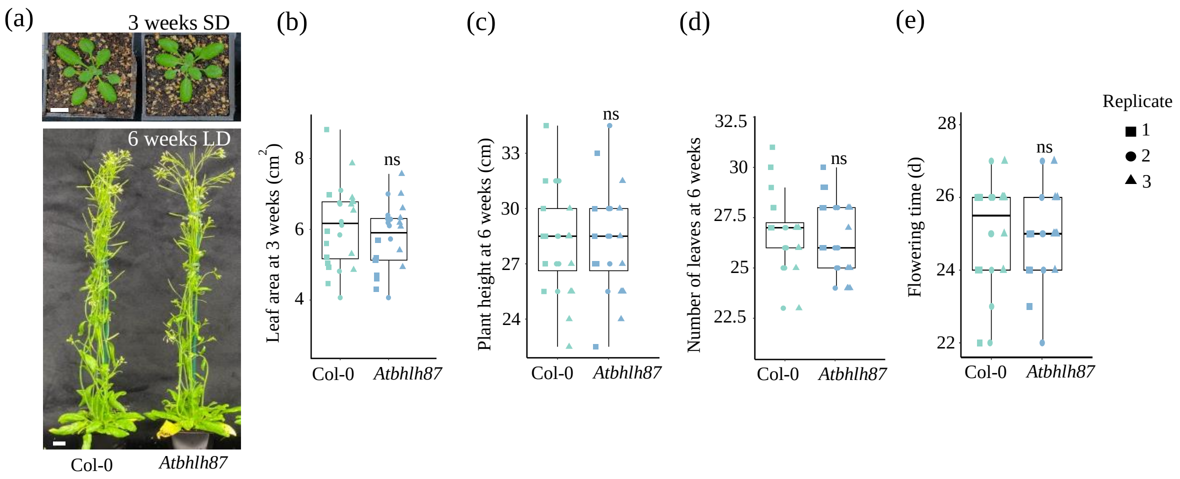


**(a)** Representative images of *Atbhlh87* mutant plants compared to wildtype (Col-0). Plants were grown under short-day (SD) or long-day (LD) conditions at 3 weeks (top) or 6 weeks (bottom) respectively. Scale bars =1 cm. (**b**) 3-week-old plants were analysed for rosette leaf area. **(c-e)** 6-week-old plants were analysed for plant height **(c)**, leaf numbers **(d)**, and flowering time **(e)**. The boxes of boxplots indicate the 25% and 75% quantiles, and the horizontal line indicates the median. The whiskers extend to the largest/smallest value no further than 1.5 times the interquartile range (n = 24). “ns” indicates no significant differences (Student's t-test).

**Figure S9.** Significantly enriched Gene Ontology (GO) terms in DEGs shared in transgenic jujube expressing *SJP39* or *ZjbHLH87*.

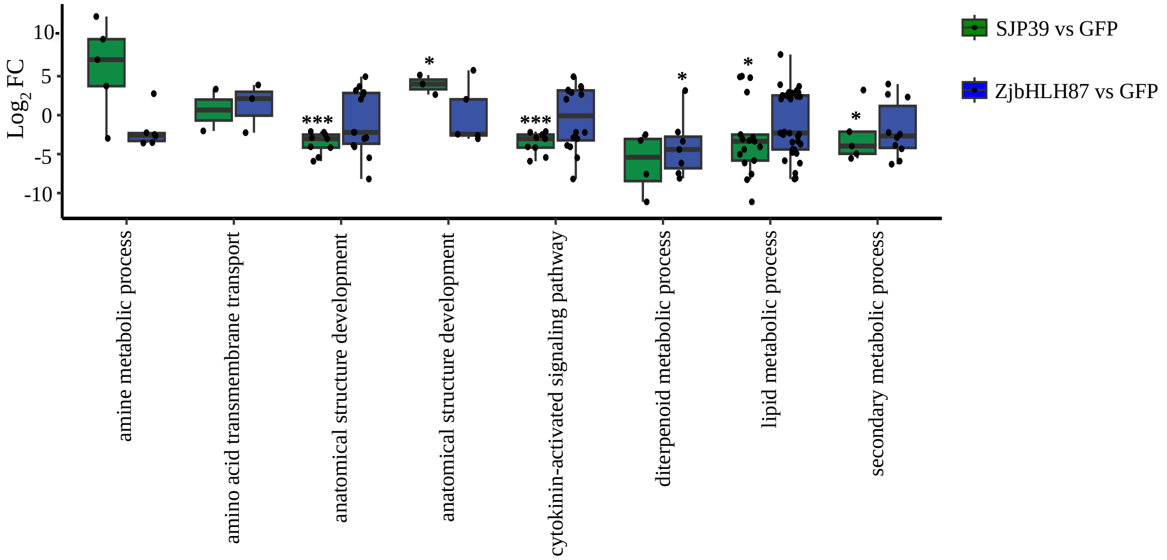


Significantly enriched genes for each GO terms were plotted based on their $log_2FC$ in SJP39 compared to GFP (green), and ZjbHLH87 compared to GFP (blue). The boxes of boxplots indicate the 25% and 75% quantiles, and the horizontal line indicates the median. The whiskers extend to the largest/smallest value no further than 1.5 times the interquartile range. Asterisks indicate $log_2FC$ values significantly different from 0 (two-tailed t test at * $p < 0.05$, ** $p < 0.01$, *** $p < 0.001$).

**Figure S10.** Expression patterns of gibberellin (GA) pathway genes in *SJP39*- and *ZjbHLH87*-expressing transgenic jujube lines.

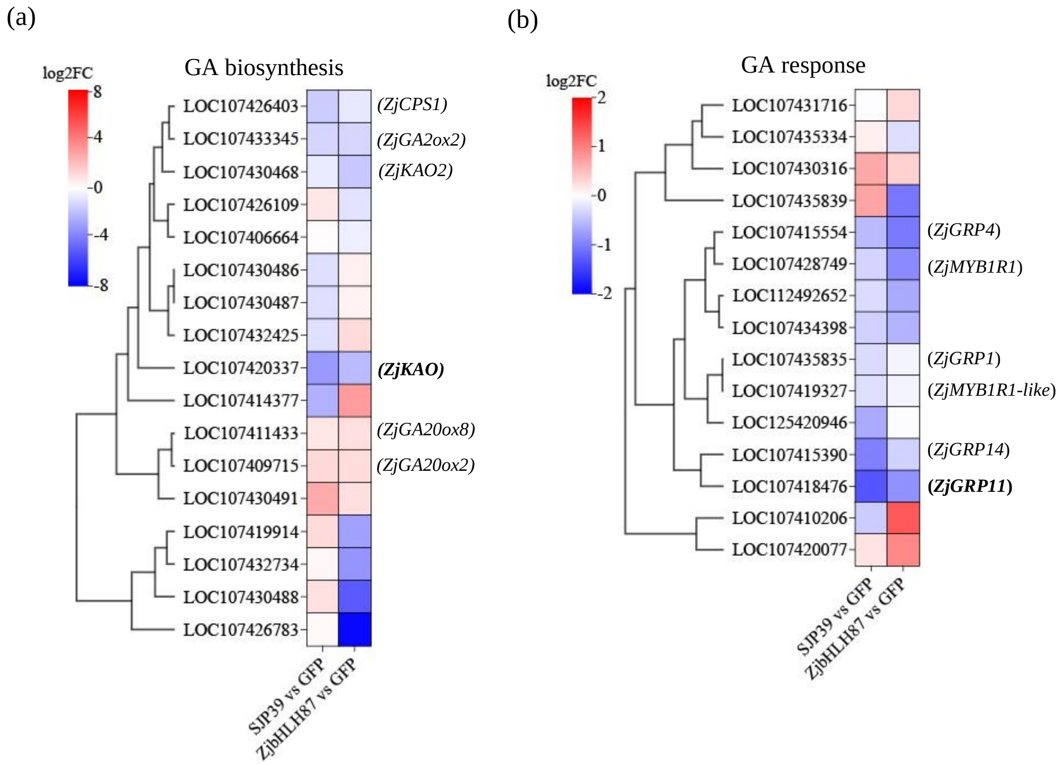


The heat map represents the $\log_2$ fold changes (log2FC) of GA biosynthesis **(a)** and response **(b)** genes in SJP39- and ZjbHLH87-expressing transgenic jujube lines compared to GFP. The data are the means of three biological replicates. Colour scale represents the signal values. *ZjCPS1: ent-copalyl diphosphate synthase 1; ZjKAO: ent-kaurenoic acid oxidase; ZjKAO2: ent-kaurenoic acid oxidase 2; ZjGA20ox2: gibberellin 20 oxidase 2. ZjGA2ox2: gibberellin 2-beta-dioxygenase 2; ZjGA20ox8: gibberellin 20 oxidase 8; ZjGRP1: gibberellin-regulated protein 1; ZjGRP4: gibberellin-regulated protein 4; ZjGRP11: gibberellin-regulated protein 11; ZjGRP14: gibberellin-regulated protein 14; ZjMYB1R1: transcription factor MYB1R1; ZjMYB1R1-like: transcription factor MYB1R1-like.*

**Figure S11**. Expression changes of genes involved in Gibberellin (GA) biosynthesis and response pathways in *SJP39*- and *ZjbHLH87*-expressing transgenic jujube lines.

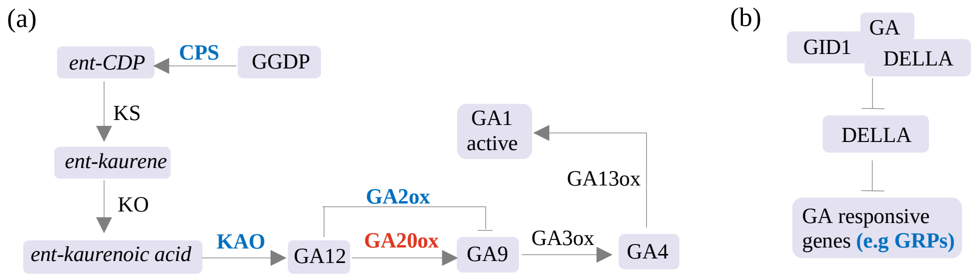


**(a)** Schematic of GA biosynthetic pathway. GAs are synthesized from geranylgeranyl diphosphate (GGDP) through a series of enzymatic reactions. Initially, GGDP is converted to ent-copalyl diphosphate (ent-CDP) by ent-copalyl diphosphate synthase (CPS), and then to ent-kaurene by ent-kaurene synthase (KS). Subsequently, ent-kaurene is oxidized to ent-kaurenoic acid by ent-kaurene oxidase (KO) and further oxidized to GA12 by ent-kaurenoic acid oxidase (KAO). GA12 is converted to GA9 by gibberellin 20 oxidase (GA20ox). The enzyme gibberellin 2-beta-dioxygenase (GA2ox) can inactivate GA12. GA9 is further converted to GA4 by gibberellin 3 oxidase (GA3ox), and GA4 is then activated to GA1 by gibberellin 13 oxidase (GA13ox). *CPS*, *KAO* and *GA2ox* were down-regulated in both *SJP39*- and *ZjbHLH87*-expressing plants while *GA20ox* was up-regulated. **(b)** Schematic of GA response pathway. Active GAs bind to the Gibberellin Insensitive Dwarf1 (GID1) and form a complex with DELLA proteins, leading to the degradation of DELLA and the subsequent activation of GA responsive genes (such as Gibberellin-regulated proteins or GRPs). *GRP*s were down-regulated in both *SJP39*- and *ZjbHLH87*-expressing plants.

**Figure S12**. Expression patterns of jasmonic acid (JA) pathway genes in *SJP39* and *ZjbHLH87* transgenic jujube lines.

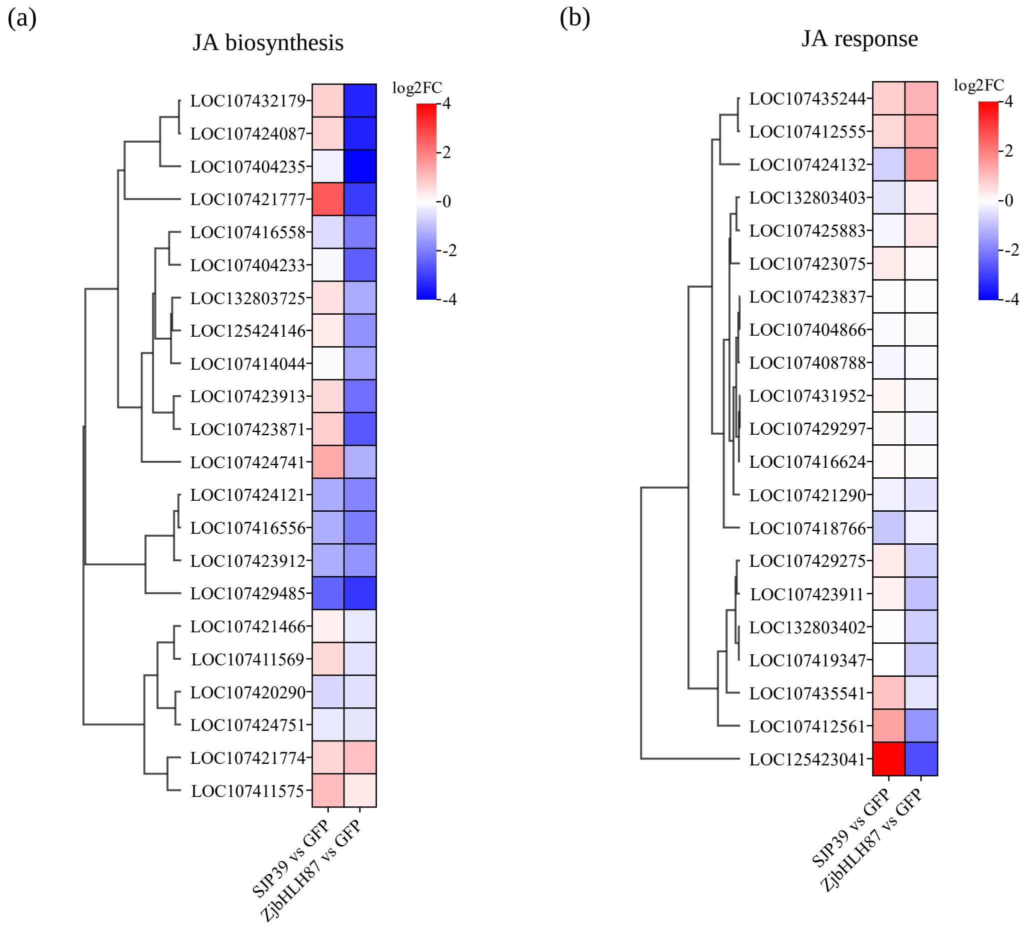


The heat map represents the log2FC of JA biosynthesis **(a)** and response **(b)** genes in transgenic plants comparing *SJP39* or *ZjbHLH87* vs *GFP*. Red and blue colours represent up- and down-regulated genes, respectively. The data are the means of three biological replicates.

**Figure S13.** Western blots confirming the expression of ZjbHLH and SJP39 in the dual-luciferase (LUC) assay.

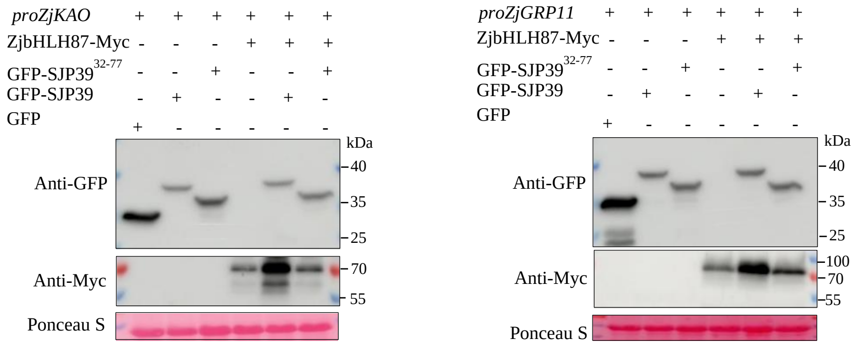


The constructs pGreen-luc-*proZjKAO* (left) or pGreen-luc-*proZjGRP11* (right) were co-expressed with ZjbHLH87-Myc, GFP, GFP-SJP39, or GFP-SJP39$^{32\text{-}77}$ in *N. benthamiana* leaves. Two days post Agroinfiltration, total proteins were extracted and analysed by western blotting using anti-GFP or anti-Myc antibodies. Ponceau S staining was used to confirm equal protein loading.
